# Divergent Small Tim Homologues Are Associated with TbTim17 and Critical for the Biogenesis of TbTim17 Protein Complexes in *Trypanosoma brucei*

**DOI:** 10.1128/mSphere.00204-18

**Published:** 2018-06-20

**Authors:** Joseph T. Smith, Ujjal K. Singha, Smita Misra, Minu Chaudhuri

**Affiliations:** a Department of Microbiology, Immunology, and Physiology, Meharry Medical College, Nashville, Tennessee, USA; University of Texas Southwestern

**Keywords:** TbTim17, *Trypanosoma brucei*, mitochondria, protein import, small Tims

## Abstract

The small Tim proteins belong to a group of mitochondrial intermembrane space chaperones that aid in the import of mitochondrial inner membrane proteins with internal targeting signals. Trypanosoma brucei, the protozoan parasite that causes African trypanosomiasis, possesses multiple small Tim proteins that include homologues of T. brucei Tim9 (TbTim9) and Tim10 (TbTim10) and a unique small Tim that shares homology with both Tim8 and Tim13 (TbTim8/13). Here, we found that these three small TbTims are expressed as soluble mitochondrial intermembrane space proteins. Coimmunoprecipitation and mass spectrometry analysis showed that the small TbTims stably associated with each other and with TbTim17, the major component of the mitochondrial inner membrane translocase in T. brucei. Yeast two-hybrid analysis indicated direct interactions among the small TbTims; however, their interaction patterns appeared to be different from those of their counterparts in yeast and humans. Knockdown of the small TbTims reduced cell growth and decreased the steady-state level of TbTim17 and T. brucei ADP/ATP carrier (TbAAC), two polytopic mitochondrial inner membrane proteins. Knockdown of small TbTims also reduced the matured complexes of TbTim17 in mitochondria. Depletion of any of the small TbTims reduced TbTim17 import moderately but greatly hampered the stability of the TbTim17 complexes in T. brucei. Altogether, our results revealed that TbTim9, TbTim10, and TbTim8/13 interact with each other, associate with TbTim17, and play a crucial role in the integrity and maintenance of the levels of TbTim17 complexes.

**IMPORTANCE** Trypanosoma brucei is the causative agent of African sleeping sickness. The parasite’s mitochondrion represents a useful source for potential chemotherapeutic targets. Similarly to yeast and humans, mitochondrial functions depend on the import of proteins that are encoded in the nucleus and made in the cytosol. Even though the machinery involved in this mitochondrial protein import process is becoming clearer in T. brucei, a comprehensive picture of protein complex composition and function is still lacking. In this study, we characterized three T. brucei small Tim proteins, TbTim9, TbTim10, and TbTim8/13. Although the parasite does not have the classical TIM22 complex that imports mitochondrial inner membrane proteins containing internal targeting signals in yeast or humans, we found that these small TbTims associate with TbTim17, the major subunit of the TbTIM complex in T. brucei, and play an essential role in the stability of the TbTim17 complexes. Therefore, these divergent proteins are critical for mitochondrial protein biogenesis in T. brucei.

## Abstract (precis)

Trypanosoma brucei is the causative agent of African sleeping sickness. The parasite’s mitochondrion represents a useful source for potential chemotherapeutic targets. Similarly to yeast and humans, mitochondrial functions depend on the import of proteins that are encoded in the nucleus and made in the cytosol. Even though the machinery involved in this mitochondrial protein import process is becoming clearer in T. brucei, a comprehensive picture of protein complex composition and function is still lacking. In this study, we characterized three T. brucei small Tim proteins, TbTim9, TbTim10, and TbTim8/13. Although the parasite does not have the classical TIM22 complex that imports mitochondrial inner membrane proteins containing internal targeting signals in yeast or humans, we found that these small TbTims associate with TbTim17, the major subunit of the TbTIM complex in T. brucei, and play an essential role in the stability of the TbTim17 complexes. Therefore, these divergent proteins are critical for mitochondrial protein biogenesis in T. brucei.

## INTRODUCTION

Trypanosoma brucei is the parasitic protist that is the causative agent of human African trypanosomiasis and nagana disease in cattle and other livestock (1–3). The parasite possesses a single, reticular mitochondrion that performs various essential functions, such as ATP production, calcium homeostasis, iron-sulfur biogenesis, and fatty acid synthesis (4–6). Similarly to the results seen with other eukaryotes, ~99% of mitochondrial proteins are nucleus encoded and need to be imported into the organelle (7, 8). Therefore, mitochondrial protein import is an essential process for the survival of the parasite. However, in spite of detailed discoveries with respect to the translocases of the mitochondrial outer and inner membranes in fungi, humans, and plants (9–12), the mitochondrial protein import machinery in trypanosomatids has just begun to be explored.

In fungi, mammals, and plants, virtually all nucleus-encoded mitochondrial proteins enter into the double-membrane organelle through the translocase of the outer membrane (TOM) complex (13, 14). Mitochondrial proteins that have an N-terminal presequence are then recognized by the TIM23 complex, which is one of two translocases of the inner membrane (TIM) (15, 16). These proteins are destined for either the mitochondrial matrix or the mitochondrial inner membrane (MIM) if they possess an additional sorting signal (17). In contrast, the TIM22 complex recognizes polytopic MIM proteins that possess internal targeting signals after translocation through the TOM complex (18–20).

Polytopic MIM proteins are hydrophobic and generally require assistance to navigate through the mitochondrial intermembrane space (IMS) to interact with the TIM22 complex (19–21). For this assistance, the small Tim chaperone complexes bind to the hydrophobic regions of the polytopic membrane proteins to prevent unnecessary protein aggregation and degradation. Among opisthokonts (fungi and mammals), there are five classical members of the small Tim protein family: Tim8, Tim9, Tim10, Tim12, and Tim13 (22, 23). In fungi, Tim9 and Tim10 form an α_3_β_3_ hexameric complex that binds to the hydrophobic regions of imported substrate proteins as they cross the TOM complex into the aqueous IMS (21, 24, 25). After the Tim9-Tim10 complex binds to its substrate protein, it then complexes with Tim12, a small Tim that is tightly associated with the TIM22 complex (26). This key interaction with Tim12 allows the substrate protein to be delivered to the TIM22 complex for integration into the MIM (27, 28). Mutations in either Tim9 or Tim10 lead to dysfunction in the import of the ADP/ATP carrier (AAC), a classical polytopic MIM protein, and to growth defects of yeast cells (20, 29). Tim8 and Tim13 form a similar, but separate, hexameric complex that has been shown to assist in the import of Tim23 and porin (21, 30, 31). However, it has also been shown that the Tim8-Tim13 complex is completely dispensable in yeast (20). Similar small Tim complexes are formed in humans, and mutations leading to a dysfunctional DDP1/Tim13 (Tim8a-Tim13) complex have been observed with the neurodegenerative disorder known as human deafness dystonia (Mohr-Tranebjærg) syndrome (32).

In spite of overall conservation in yeast, human, and plants, mitochondrial protein import machinery in trypanosomatid parasites is significantly divergent (33–36). T. brucei possesses a noncanonical TOM complex, known as the archaic translocase of the mitochondrial outer membrane (ATOM) complex (34, 35, 37). The parasite also possesses a unique TIM complex consisting of T. brucei Tim17 (TbTim17) and several other trypanosome-specific proteins that associate with TbTim17 (36, 38, 39). TbTim17 is the single member of the Tim17/Tim22/Tim23 protein family that is found in T. brucei. Furthermore, no homologues for any of the components of the fungal TIM22 complex have been detected *in silico* in trypanosomatids. However, in spite of the absence of a recognizable TIM22 complex in T. brucei, the parasite expresses at least five small Tims, referred as TbTim9, TbTim10, TbTim11, and TbTim13, and one small Tim that shows homology to fungal Tim8 and Tim13 and that has thus been named TbTim8/13. In addition, there is a small Tim-like protein in trypanosomatids, TbTim12 (23, 35, 39). Recently, TbTim11, TbTim12, and TbTim13 have been further characterized (40), but the roles of TbTim9, TbTim10, and TbTim8/13 in mitochondrial protein import have not been explored. Here we found that these small TbTims are soluble mitochondrial intermembrane space proteins but that they are stably associated with TbTim17. These small TbTims are also able to directly interact with each other and play a crucial role in the biogenesis and stability of the TbTim17 complexes in procyclic T. brucei parasites.

## RESULTS

### TbTim9, TbTim10, and TbTim8/13 have divergent amino acid sequences but possess tertiary structures that are similar to those of small Tims in other species, as well as a conserved twin CX_3_C motif.

**FIG 1:**
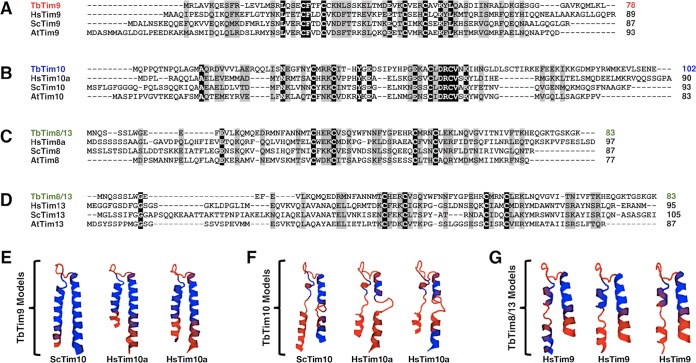
Bioinformatics analysis of the small TbTim amino acid sequences. **(**A to D) Clustal Omega multiple-sequence alignment of the primary amino acid sequences of (A) TbTim9, HsTim9, ScTim9, and AtTim9; (B) TbTim10, HsTim10, ScTim10, and AtTim10; (C) TbTim8/13, HsTim8a, HsTim8, ScTim8, and AtTim8; and (D) TbTim8/13, HsTim13, HsTim13, ScTim13, and AtTim13 (ScTim, Saccharomyces cerevisiae Tim; HsTim, Homo sapiens Tim; AtTim, Arabidopsis thaliana Tim). Identical amino acid residues are highlighted in black; similar amino acid residues are highlighted in gray. (E to G) Protein structure homology modeling using the SWISS-MODEL ExPASy Web server based on available crystal structures. The PDB numbers for the crystal structures used as templates are as follows: ScTim10, 3dxr.1.B; HsTim10a, 2bsk.1.B; HsTim9, 2bsk.1.A. The top three predicted tertiary structure models of (E) TbTim9, (F) TbTim10, and (G) TbTim8/13 are shown from most probable best fit to least probable best fit from left to right. The small Tim homologue that was used as the template is shown below the corresponding best-fit model. All protein models are shown with the N terminus on the left and the C terminus on the right. Blue indicates regions of conserved tertiary structure, and orange indicates regions of divergent tertiary structure.

T. brucei expresses at least 5 small TbTims. Among these, three small TbTims, TbTim9, TbTim10, and TbTim8/13, were initially identified by Gentle et al., in 2007, using hidden Markov model-based homology searches in the trypanosome genome databases (23). BLAST analysis showed that TbTim9, TbTim10, and TbTim8/13 possess overall 9% to 26% identity and 24% to 42% similarity, whereas fungal and human small Tims are ~33% identical and >50% similar in their primary sequences (see Table S1 in the supplemental material). Multiple-sequence alignments performed in different combinations using the Clustal Omega program (41) revealed that most of the amino acid similarity and identity appeared centralized within the small TbTims, while the N and C termini were relatively divergent (Fig. 1A to D). Each of the small TbTims possesses the twin CX_3_C motif that was aligned well with similar motifs in other small Tims from human and fungi (Fig. 1A to D).

10.1128/mSphere.00204-18.7 TABLE S1  Amino acid similarity and identity between small Tim homologues. The primary amino acid sequences of the T. brucei, yeast, plant, or human small Tim proteins were entered in the EMBOSS Needle pairwise sequence alignment software. The amino acid similarities and identities are shown as percentages. The T. brucei small Tims (TbTim) were compared with their yeast (ScTim), plant (AtTim), and human (HsTim) counterparts individually. Human-plant and human-yeast comparisons (in bold) are also shown as reference points. The small TbTims were also compared to one another. TbTim9 was compared to Tim9 homologues; TbTim10 was compared to Tim10 homologues; TbTim8/13 was compared to Tim8 and Tim13 homologues. Download TABLE S1, PDF file, 0.3 MB. Copyright © 2018 Smith et al. 2018 Smith et al. This content is distributed under the terms of the Creative Commons Attribution 4.0 International license.

We also compared the predicted tertiary structures of the small TbTims by using the SWISS-MODEL ExPASy Web server (42–45). The amino acid primary sequence of the small TbTims was entered into the SWISS-MODEL server with no proposed protein template to obtain the most probable best-fit tertiary structure models for the small TbTims based on available crystal structures, according to their global model quality estimate (GMQE) scores (Fig. 1E to G). Typical small Tim proteins have N-terminal and C-terminal α-helices separated by a short random coil region (46). The two α-helices are connected by two intramolecular disulfide bonds formed by the cysteine residues of the twin CX_3_C motif (47). The predicted models for TbTim9 (GMQE scores ranging from 0.57 to 0.61) showed well-conserved tertiary small Tim structures, with only the extreme N- and C-terminal regions showing structural divergence (Fig. 1E). Interestingly, TbTim9 appeared to be structurally closer to yeast and human Tim10 proteins than to yeast and human Tim9 proteins, in contrast to our initial expectations (Fig. 1E). TbTim10 was shown to also be closest in structure to other Tim10 homologues (GMQE scores ranging from 0.44 to 0.46), but the predicted structures were not tightly conserved, as the N- and C-terminal helices appeared to be loosely folded (Fig. 1F). Surprisingly, the predicted protein models for TbTim8/13 (GMQE scores ranging from 0.35 to 0.40) were closest to neither the Tim8 homologues nor the Tim13 homologues but rather were closest in structure to human Tim9 (Fig. 1G). In all TbTim8/13 models, the N-terminal α-helix was not completely folded, and the extreme C terminus was divergent (Fig. 1G). Moreover, in all small TbTim homology models, the two pairs of cysteine residues of the twin CX_3_C motif were juxtaposed in close proximity (see Fig. S1 in the supplemental material), which could imply the potential for intramolecular disulfide bonds in these proteins. Therefore, these data imply that the small TbTims may form tertiary structures that are somewhat similar to those of yeast and human small Tims, despite the divergence in their amino acid sequences.

10.1128/mSphere.00204-18.1 FIG S1  Homology modeling of the small TbTim twin CX_3_C motifs. Using the top half of the protein structure homology models shown in Fig. 1E to G, only the cysteine residues in the twin CX_3_C motifs are shown by three-dimensional atomic stick models within the original ribbon models of the predicted tertiary structures. The three homology models for **(**A to C) TbTim9, (D to F) TbTim10, and (G to I) TbTim8/13 are displayed here twice: once with the inner cysteines of the twin CX_3_C motif (C2 and C3, left model) and once with the outer cysteines of the twin CX_3_C motif (C1 and C4, right model). The models are presented in the same order from left to right as shown in Fig. 1E to G. Download FIG S1, PDF file, 1.1 MB. Copyright © 2018 Smith et al. 2018 Smith et al. This content is distributed under the terms of the Creative Commons Attribution 4.0 International license.

### The small TbTims are soluble mitochondrial proteins.

**FIG 2:**
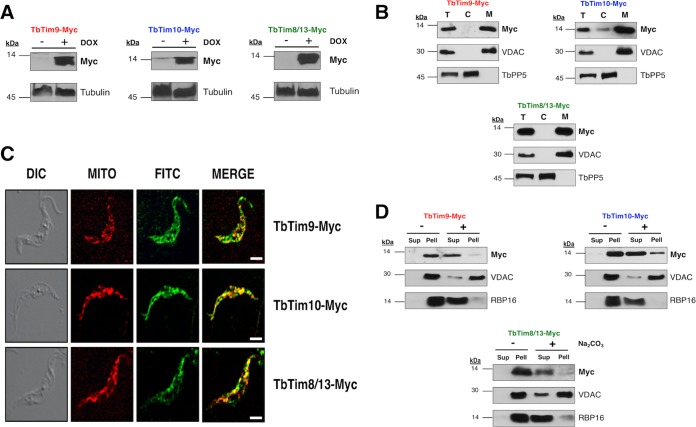
Expression and mitochondrial localization of the small TbTim proteins. (A) Inducible expression of TbTim9-Myc, TbTim10-Myc, and TbTim8/13-Myc in T. brucei. Cells were grown in the absence (−) and presence (+) of doxycycline (DOX) for 48 h, and total cellular proteins were analyzed by SDS-PAGE and immunoblot analysis using anti-myc monoclonal antibody. Tubulin was used as a loading control. (B) Subcellular fraction of T. brucei cells expressing TbTim9-Myc, TbTim10-Myc, or TbTim8/13-Myc. Proteins from the total cell lysates (lanes T), cytosolic fractions (lanes C), and mitochondrial fractions (lanes M) were subjected to SDS-PAGE and were probed with anti-myc antibody. VDAC and TbPP5 were used as the mitochondrial and cytosolic protein markers, respectively. (C) *In situ* immunofluorescence staining of TbTim9-Myc, TbTim10-Myc, and TbTim8/13-Myc cells. Live cells were stained with MitoTracker Red and then stained with anti-myc antibody as the primary antibody and FITC-conjugated anti-mouse IgG as the secondary antibody. Confocal microscopy images were taken using an LSM510 confocal microscope at ×60 magnification. The differential interference contrast (DIC), MitoTracker Red (MITO), and FITC images were merged to show colocalization. Size bar, 5 µm. (D) Alkaline sodium carbonate extraction of mitochondria isolated from T. brucei cells expressing the ectopic small TbTim proteins. Mitochondria were treated (+) with 100 mM sodium carbonate (pH 11.5) or left untreated (−) on ice for 30 min. Soluble (Sup) and insoluble (Pell) fractions were separated by centrifugation, and proteins were analyzed by Western blotting using anti-myc antibodies. VDAC and RBP16 were used as markers for membrane-bound and soluble proteins, respectively.

We generated procyclic T. brucei cell lines that inducibly express a C-terminally 2×-myc-tagged copy of the small TbTims (TbTim9-Myc, TbTim10-Myc, and TbTim8/13-Myc). Analysis of total cellular proteins after induction of cells with doxycycline (1.0 µg/ml) for 48 h by SDS-PAGE followed by immunodecoration with anti-myc antibody revealed that the small TbTims were expressed at the predicted sizes for TbTim9-Myc (11 kDa), TbTim10-Myc (14 kDa), and TbTim8/13-Myc (12 kDa) in their respective cell lines after induction with doxycycline (Fig. 2A). Small TbTim overexpression did not have any effect on cell growth (Fig. S2).

10.1128/mSphere.00204-18.2 FIG S2  Effect of small TbTim overexpression on T. brucei cell growth. Cell growth analysis of T. brucei parasites that express (A) TbTim9-Myc, (B) TbTim10-Myc, or (C) TbTim8/13-Myc in the absence (uninduced) or presence (induced) of doxycycline (1.0 µg/ml) for 6 days. The log of the cumulative cell numbers was plotted versus time (in days). Download FIG S2, PDF file, 1.2 MB. Copyright © 2018 Smith et al. 2018 Smith et al. This content is distributed under the terms of the Creative Commons Attribution 4.0 International license.

In order to determine if the ectopic small TbTims were properly localized in T. brucei mitochondria, we performed subcellular fractionation to separate the mitochondrial fraction from the cytosolic fraction of TbTim9-Myc, TbTim10-Myc, and TbTim8/13-Myc cells. Immunoblot analysis using the anti-myc antibody showed that the ectopic small TbTim proteins were primarily localized in the mitochondrial fraction (Fig. 2B). The voltage-dependent anion channel protein (VDAC) (48) was used as a mitochondrial protein control, and T. brucei protein phosphatase 5 (TbPP5) (49) was used as a cytosolic marker. *In situ* immunofluorescence microscopy was also performed using an anti-myc primary antibody and a fluorescein isothiocyanate (FITC)-conjugated secondary antibody to locate the ectopically expressed small TbTim proteins in T. brucei cells (Fig. 2C). Before antibody staining, live cells were treated with MitoTracker Red to stain the parasite mitochondrion. Colocalization of the FITC (green) and MitoTracker (red) staining showed that TbTim9-Myc, TbTim10-Myc, and TbTim8/13-Myc proteins were localized in T. brucei mitochondria. These results further confirmed our cell fractionation data.

Next, we investigated if the mitochondrial small TbTim proteins were soluble in the mitochondrion or membrane integrated. We solubilized mitochondria isolated from T. brucei cells that expressed TbTim9-Myc, TbTim10-Myc, or TbTim8/13-Myc in alkaline sodium carbonate buffer and centrifuged the lysate to separate the membrane proteins in the pellet from the soluble proteins in the supernatant. Immunoblot analysis performed with the anti-myc antibody revealed that the ectopic small TbTims were soluble proteins (Fig. 2D). VDAC was used as a membrane protein control (48), and RNA-binding protein 16 (RBP16), which is a mitochondrial matrix protein (50), was used as a soluble protein control, as previously demonstrated (51).

### The small TbTims are in the mitochondrial intermembrane space.

**FIG 3:**
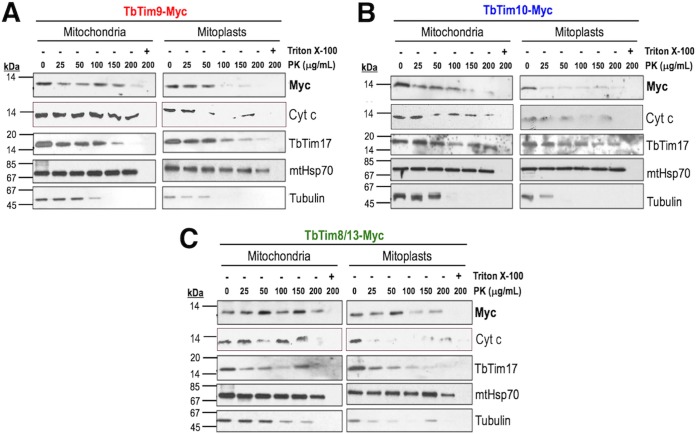
Submitochondrial localization of the small TbTim proteins. Proteinase K protection assays of mitochondria (left panels) and mitoplasts (right panels) isolated from T. brucei cells expressing (A) TbTim9-Myc, (B) TbTim10-Myc, or (C) TbTim8/13-Myc were performed. Mitochondria and mitoplasts were treated with various concentrations (0 to 200 µg/ml) of PK as described in Materials and Methods. Proteins were analyzed by SDS-PAGE and immunoblot analysis using anti-myc, anti-Cyt *c*, anti-TbTim17, anti-mtHsp70, and anti-tubulin antibodies.

Proteinase K (PK) protection assays were performed to verify the submitochondrial localization of the ectopic small TbTims. We found that the small TbTims were protected when mitochondria were treated with PK at concentrations up to 100 to 150 µg/ml (Fig. 3). However, when mitochondria were swelled to generate mitoplasts before PK digestion, the small TbTims were mostly digested at lower concentrations (25 to 50 µg/ml) of PK (Fig. 3). We also noticed that in mitoplasts, TbTim9 and TbTim10 were more resistant than TbTim8/13 to PK digestion. This could be because the protease-sensitive regions of TbTim9 and TbTim10 are exposed in the IMS and the similar region of TbTim8/13 may be covered by other proteins (Fig. S3). A similar phenomenon was observed where Tim10 in yeast was resistant to the Yme1 quality control protease, as well as trypsin, due to protection from Tim9 (29). Therefore, a similar scenario may be present in T. brucei that renders some of the small TbTims less sensitive to protease digestion. As expected, cytochrome *c* (Cyt *c*), an IMS protein, was also degraded at a lower concentration of PK when the mitochondrial outer membrane (MOM) was ruptured, but Cyt *c* was mostly protected from protease digestion of mitochondria (Fig. 3). In contrast, mtHsp70, a mitochondrial matrix protein, was completely protected from PK digestion in mitochondria and mitoplasts. TbTim17, a MIM protein, was protected from PK digestion in mitochondria. However, it was partially digested when mitoplasts were treated with PK (>100 µg/ml). In contrast, tubulin, a protein that is peripherally associated with the MOM, showed sensitivity to PK concentrations of ≥50 µg/ml in mitochondria and ≥25 µg/ml in mitoplasts. All proteins were digested with PK when mitochondrial membranes were solubilized with 1% Triton X-100. These results showed that the small TbTims are localized in the IMS. Altogether, the data suggested that the ectopic small TbTim proteins were targeted to mitochondria and exist as soluble IMS proteins in T. brucei, similarly to other eukaryotes.

10.1128/mSphere.00204-18.3 FIG S3  Quantitation of immunoblots for mitochondrial proteins after osmotic swelling and proteinase K treatment. The signal intensities of Myc, Cyt *c*, TbTim17, and tubulin intensities detected in immunoblots are normalized to the mtHsp70 signal intensities in **(**A and B) TbTim9-Myc mitochondria and mitoplasts, (C and D) TbTim10-Myc mitochondria and mitoplasts, and (E and F) TbTim8/13-Myc mitochondria and mitoplasts. Signal intensities detected in the respective mitochondrial samples (0 µg/ml·PK) were set to 100%. Download FIG S3, PDF file, 1.9 MB. Copyright © 2018 Smith et al. 2018 Smith et al. This content is distributed under the terms of the Creative Commons Attribution 4.0 International license.

### The small TbTims assemble into larger protein complexes and associate with TbTim17.

**FIG 4:**
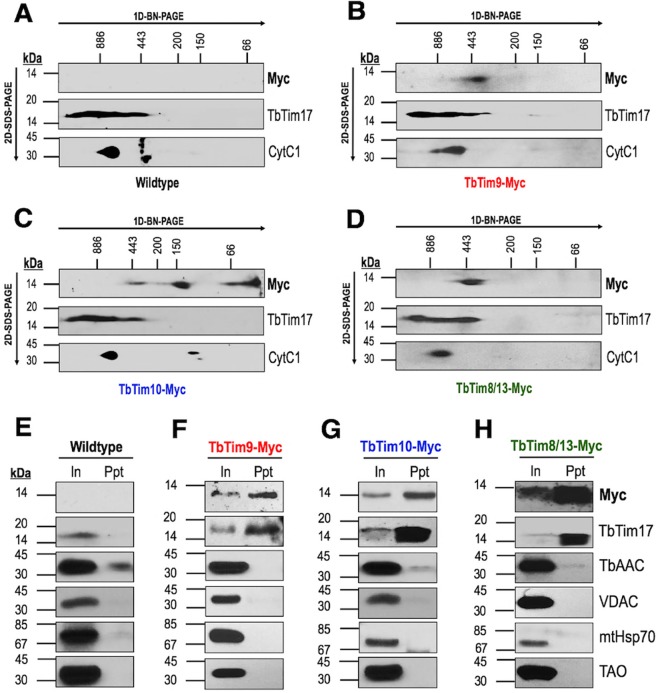
The small TbTims are associated with TbTim17. **(**A to D) Two-dimensional blue native/SDS-PAGE of digitonin-solubilized mitochondrial extracts from (A) wild-type, (B) TbTim9-Myc, (C) TbTim10-Myc, and (D) TbTim8/13-Myc cells followed by immunoblot analysis performed with anti-myc, anti-TbTim17, and anti-CytC1 antibodies. Respective molecular weight markers for the blue native gels (1D-BN-PAGE) and SDS-PAGE gels (2D–SDS-PAGE) are shown. (E to H) Coimmunoprecipitation of digitonin-solubilized mitochondrial extracts from (E) wild-type, (F) TbTim9-Myc, (G) TbTim10-Myc, and (H) TbTim8/13-Myc cells using agarose beads coupled with the anti-myc antibody. Bound proteins were analyzed on SDS-PAGE followed by immunoblot analysis using antibodies for the myc epitope, TbTim17, TbAAC, VDAC, mtHsp70, and TAO. Input (In) lanes were loaded with 10% (vol/vol) of total solubilized proteins; precipitate/bound (Ppt) lanes were loaded with 50% (vol/vol) of the total pulldown fraction. Experiments were repeated three times, and representative results from one trial are shown.

Solubilized protein complexes of mitochondria from wild-type, TbTim9-Myc, TbTim10-Myc, and TbTim8/13-Myc cells were analyzed by two-dimensional blue native (BN)/SDS-PAGE (2D-PAGE) followed by immunoblot analysis using anti-myc, anti-TbTim17, and anti-cytochrome *bc*-1 (CytC1) antibodies. As expected, the anti-myc antibody did not recognize any proteins from wild-type mitochondria (Fig. 4A). Using the same antibody, we found that TbTim9-Myc and TbTim8/13-Myc were both assembled into a large (~400-kDa) complex (Fig. 4B and D), while TbTim10-Myc was present in complexes of three different sizes (<66 kDa, ~150 kDa, and ~400 kDa) as detected by the anti-myc antibody (Fig. 4C). The sizes of the larger complexes overlapped the sizes of TbTim17 complexes (Fig. 4B to D). The anti-TbTim17 and anti-CytC1 antibodies detected their respective protein complexes, as described in our previous report (38), thus verifying the integrity of the mitochondrial protein complexes after solubilization.

To verify if the small TbTims associated with TbTim17, a coimmunoprecipitation experiment was performed. Coimmunoprecipitated proteins were subjected to immunoblot analysis using anti-TbTim17 and anti-myc antibodies. Blots were also probed with antibodies for other mitochondrial proteins as controls. Anti-myc antibody did not precipitate any proteins from the wild-type mitochondrial extract, as expected (Fig. 4E). However, our results clearly showed that TbTim17 was coimmunoprecipitated with TbTim9-Myc, TbTim10-Myc, and TbTim8/13-Myc by anti-myc immunoprecipitation pulldown (Fig. 4F to H). Other mitochondrial proteins, such as TbAAC, VDAC, mtHsp70, or TAO, were not detected in the precipitated fraction by immunoblot analysis. Together, these results confirmed that TbTim17 is associated with these small TbTim proteins. Furthermore, mass spectrometry (MS) analysis of the immunoprecipitate from the solubilized TbTim10-Myc mitochondrial extract identified TbTim9, TbTim10, TbTim8/13, TbTim11, TbTim12, TbTim13, TbTim17, and other TbTim17-associated proteins (Table S2) (a complete list of the identified proteins is presented in Table S3). Mass spectrometry analysis also identified VDAC and Atom40, two β-barrel MOM proteins, as well as TbAAC in the immunoprecipitate, although these were not detected by immunoblot analysis. These proteins are the substrates for small Tim chaperones in other eukaryotic mitochondria (18). Therefore, association of TbTim10 with these proteins may suggest similar functions for the small TbTims in T. brucei mitochondria. As reported recently (40), our data showed that all small TbTims are associated with each other and also with the TbTim17 complexes in T. brucei.

10.1128/mSphere.00204-18.8 TABLE S2  A partial list of proteins identified from the coimmunoprecipitated samples by mass spectrometry analysis. Proteins were immunoprecipitated by the use of anti-myc-coupled agarose beads from the mitochondrial lysate obtained from the parental (wild-type) T. brucei cells and from the cells expressing TbTim10-Myc. Bound proteins were analyzed on an SDS-PAGE gel and stained with Coomassie blue. Lanes for individual samples were excised from the gel, proteins were digested by trypsin, and peptides were analyzed by mass spectrometry. A complete list of the identified proteins is presented in Table S3. Download TABLE S2, PDF file, 1.1 MB. Copyright © 2018 Smith et al. 2018 Smith et al. This content is distributed under the terms of the Creative Commons Attribution 4.0 International license.

10.1128/mSphere.00204-18.9 TABLE S3  Complete list of the identified proteins. Download TABLE S3, XLSX file, 0.02 MB. Copyright © 2018 Smith et al. 2018 Smith et al. This content is distributed under the terms of the Creative Commons Attribution 4.0 International license.

### TbTim8/13 interacts directly with TbTim9 and TbTim10.

**FIG 5:**
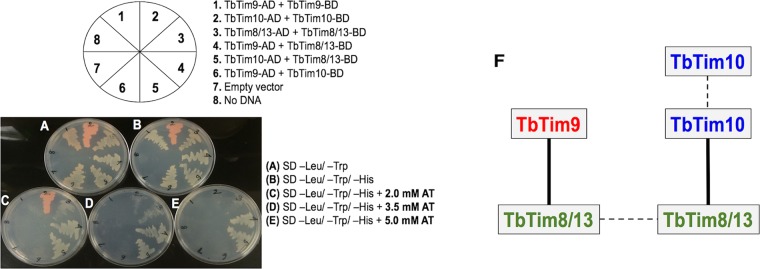
Yeast two-hybrid analysis of direct interactions of the small TbTims. Representative plates showing yeast cotransformed with the small TbTim pGADT7 (activation domain, AD) and pGBKT7 (DNA-binding domain, BD) plasmids were grown in (A) synthetic-defined (SD) medium lacking leucine and tryptophan (–leu/–trp), (B) medium lacking leucine, tryptophan, and histidine (–leu/–trp/–his), (C) –leu/–trp/–his medium containing 2.0 mM 3-amino-1,2,4-triazole (AT), (D) –leu/–trp/–his medium containing 3.5 mM AT, and (E) –leu/–trp/–his medium containing 5.0 mM AT. The schematic of small TbTim AD and BD plasmid combinations for cotransformation is shown. Yeast cells cotransformed with empty vector (pGADT7) or no DNA were used as negative controls. The plates shown here were obtained from one of three independent experiments. (F) A schematic of the small TbTim interaction pattern. Solid lines represent a stronger interaction, and dotted lines represent a weaker interaction.

We used yeast two-hybrid analysis to determine if the small TbTims directly interact with one another. For this purpose, TbTim9, TbTim10, and TbTim8/13 were cloned in the bait (pGBKT7) and prey (pGADT7) plasmids to be expressed in yeast as fusion proteins with the DNA-binding domain (BD) or activation domain (AD) of the GAL4 transcription factor. Fusion proteins are expressed with a C-terminal Myc or hemagglutinin (HA) epitope tag from the pGBKT7 or pGADT7 plasmid vector, respectively. Positive cotransformants were selected in synthetically defined (SD) medium lacking leucine and tryptophan (–leu/–trp medium) (Fig. 5A). To test the small TbTim interactions, we grew positive cotransformants in SD medium lacking leucine, tryptophan, and histidine (–leu/–trp/–his medium) in various concentrations (0 to 5 mM) of 3-amino-1,2,4-triazole (AT) (Fig. 5B to E) to reduce false positives. The cotransformants that grew only at lower AT concentrations (2.0 mM and 3.5 mM), such as TbTim10-AD and TbTim10-BD (section 2 in Fig. 5A) and TbTim8/13-AD and TbTim8/13-BD (section 3), were considered weakly interacting partners (Fig. 5C and D). Cotransformants that grew robustly even at 5.0 mM AT, such as TbTim9-AD plus TbTim8/13-BD (section 4 in Fig. 5A) and TbTim10-AD plus TbTim8/13-BD (section 5), showed strong interaction with their partner proteins (Fig. 5E). Yeast cells cotransformed with plasmid pair TbTim9-AD plus TbTim9-BD (section 1 in Fig. 5A) and plasmid pair TbTim9-AD plus TbTim10-BD (section 6) grew only in the absence of AT, showing that the homotypic association of TbTim9 and the interaction between TbTim9 and TbTim10 are very weak. As expected, yeast cells cotransformed with empty pGADT7 and pGBKT7 plasmids did not grow in SD –leu/–trp/–his plates without or with AT (Fig. S4A to D). The parental yeast cells that were transformed with an empty pGADT7 plasmid (section 7 in Fig. 5A) or received no plasmid DNA (section 8) did not grow even in SD –leu/–trp plates, as expected. We observed reddish coloration of some yeast colonies. The adenine-deficient yeast mutants used in this study accumulate an intermediate that often oxidizes during aerobic growth and sometimes produces red colonies that do not have any correlation with the interaction of the partner proteins. We also verified that the small TbTim fusion proteins were expressed in transformed yeast cells (Fig. S4E). Taking the data together, TbTim8/13 interacts strongly with TbTim9 as well as with TbTim10; however, the interaction between TbTim9 and TbTim10 is very weak (Fig. 5F).

10.1128/mSphere.00204-18.4 FIG S4  Expression of the fusion proteins TbTim9-Gal4BD, TbTim10-Gal4BD, and TbTim8/13-Gal4BD in yeast. **(**A to D) Yeast cell cotransformed with the empty pGADT7 and pGDKT7 plasmid constructs were grown in synthetic defined (SD) –trp/–leu medium or –trp/–leu/–his medium containing 0 to 5 mM AT to show that yeast cells cotransformed with empty plasmids are unable to grow in triple-knockout medium. (E) A Saccharomyces cerevisiae strain (Y2H Gold) transformed with the TbTim9, TbTim10, and TbTim8/13 fusion constructs in the pGDKT7 plasmid was grown in synthetic defined (SD) medium lacking tryptophan (–trp). The pGDKT7 plasmid adds a Myc epitope to Gal4BD. Cells (optical density [OD] of 3) were pelleted by centrifugation, resuspended in 200 µl of 0.1 M NaOH, and incubated at room temperature for 5 min. Cells were recovered by centrifugation and lysed with reducing Laemmli buffer (300 µl), and soluble proteins were subjected to immunoblot analysis using anti-myc antibody. A 20-μl volume of soluble proteins was loaded per lane. A Y2H strain that was not transfected with any plasmid was grown in yeast extract-peptone-dextrose-adenine (YPDA) medium, processed similarly, and used as a control. A nonspecific band present in all lanes is indicated by an asterisk (*). Download FIG S4, PDF file, 0.1 MB. Copyright © 2018 Smith et al. 2018 Smith et al. This content is distributed under the terms of the Creative Commons Attribution 4.0 International license.

### Small TbTim knockdown reduces T. brucei cell growth.

**FIG 6:**
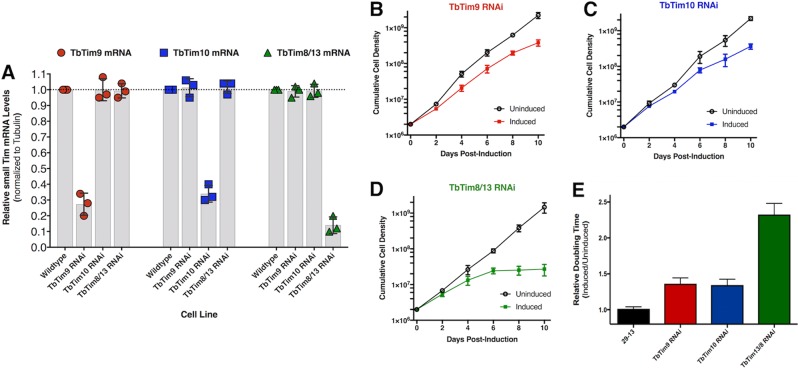
Effect of small TbTim knockdown on cell growth. (A) Scatterplot representation of qRT-PCR analysis of TbTim9, TbTim10, and TbTim8/13 mRNA levels in wild-type, TbTim9 RNAi, TbTim10 RNAi, and TbTim8/13 RNAi T. brucei cells after 96 h of induction with doxycycline (1.0 µg/ml). The mRNA levels of the target transcript in wild-type parasites were considered to represent 100%. Small TbTim transcript levels were normalized with the levels of the tubulin mRNA in each sample. Values were calculated from three independent replicates (****, *P* < 0.0001) and are represented as scatterplots with error bars for standard deviations. (B to D) Cell growth analysis of small TbTim RNAi parasites in the absence (uninduced) and presence (induced) of doxycycline (1.0 µg/ml) for 10 days. The log of the cumulative cell numbers was plotted versus time. (E) Doubling time of TbTim9 RNAi, TbTim10 RNAi, and TbTim8/13 RNAi in the absence (uninduced) and presence (induced) of doxycycline from day 2 to day 8. Data are shown as relative ratios of induced/uninduced results. The 29-13 cell line was used for comparisons.

TbTim9 RNA interference (RNAi), TbTim10 RNAi, and TbTim8/13 RNAi procyclic cell lines were allowed to grow in the absence and presence of doxycycline (1 µg/ml) for induction of expression of the double-stranded RNA. Quantitative reverse transcriptase PCR (qRT-PCR) analysis showed that in comparison to the wild-type control, the levels of each small TbTim transcript were significantly reduced in the respective RNAi cell lines after induction with doxycycline (Fig. 6A) whereas the small TbTim transcript levels were similar among the wild-type and uninduced RNAi cells, as expected (Fig. S5A). Cell growth analysis revealed that knockdown of TbTim9 and TbTim10 caused a noticeable reduction in the rate of postinduction cell growth as early as day 4 and after (Fig. 6B to D). Moreover, the doubling time of induced small TbTim RNAi markedly increased (Fig. 6E). Interestingly, TbTim8/13 knockdown also reduced cell growth and caused cell proliferation to cease at day 6 postinduction (Fig. 6D), indicating that TbTim8/13 is essential for cell growth. This is in agreement with Gentle et al., who had also shown that Tim8/13 knockdown was detrimental to T. brucei cell growth (23).

10.1128/mSphere.00204-18.5 FIG S5  Wild-type and uninduced RNAi control results were comparable. (A) Scatterplot representation of quantitative reverse transcriptase PCR (qRT-PCR) analysis of TbTim9, TbTim10, and TbTim8/13 mRNA levels in wild-type, TbTim9 RNAi, TbTim10 RNAi, and TbTim8/13 RNAi T. brucei cells after 96 h either in the absence (uninduced RNAi) or the presence (induced RNAi) of doxycycline (1.0 µg/ml). The mRNA levels of the target transcript in wild-type parasites were considered to represent 100%. Small TbTim transcript levels were normalized with the levels of the tubulin mRNA in each sample. Values were calculated from three independent replicates (****, *P* < 0.0001), and error bars indicate standard deviations. (B) Mitochondrial protein analyzed by immunoblotting to determine the steady-state levels of TbTim17, TbAAC, and TAO. Amounts of proteins (50 µg and 25 µg) loaded for each sample are indicated by the right-pointing triangles. Download FIG S5, PDF file, 0.1 MB. Copyright © 2018 Smith et al. 2018 Smith et al. This content is distributed under the terms of the Creative Commons Attribution 4.0 International license.

### Small TbTim knockdown significantly reduces the steady-state levels of TbTim17.

**FIG 7:**
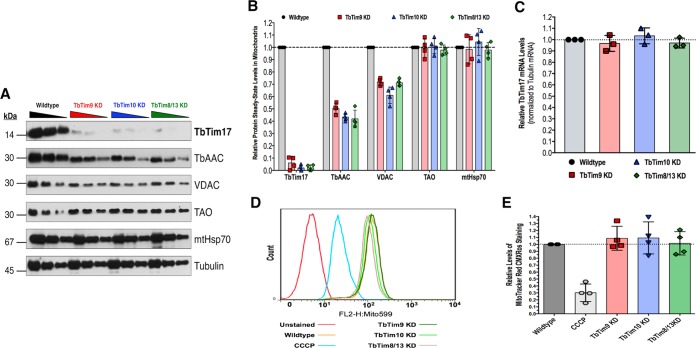
Mitochondrial protein profile of small TbTim knockdown (KD) mitochondria. (A) Western blot analysis of mitochondria (50 µg, 25 µg, and 12.5 µg in decreasing order as indicated by the triangular symbol) isolated from wild-type, TbTim9 RNAi (TbTim9 KD), TbTim10 RNAi (TbTim10 KD), and TbTim8/13 RNAi (TbTim8/13 KD) T. brucei cells grown in the presence of doxycycline (1.0 µg/ml) for 96 h using antibodies as indicated. (B) Scatterplot representation of densitometric analysis of the immunoblots using ImageJ software. The intensities of the indicated protein signals for wild-type mitochondria were normalized to the tubulin signal (loading control). The normalized values for the wild-type sample were set to 100% (1.0). Error bars indicate standard deviations of results from four independent experiments. (C) Scatterplot representation of qRT-PCR analysis of the levels of TbTim17 transcript in the wild-type and small TbTim RNAi cells. Tubulin was used as the internal control. The relative levels of TbTim17 mRNA in small TbTim RNAi cells were calculated by considering the levels in wild-type parasites to represent 1.0. Values were calculated from three independent replicates. Error bars indicate standard deviations. (D) Effect of small TbTim knockdown on MitoTracker Red staining. Cells were treated with doxycycline for 96 h, stained with MitoTracker Red, and fixed with 0.37% paraformaldehyde, and fluorescence intensity was measured at 599 nm by fluorescence-activated cell sorter (FACS) analysis. Wild-type cells pretreated with 50 µM CCCP for 15 min before MitoTracker Red staining served as a positive control for depleted mitochondrial membrane potential. (E) Scatterplot representation of quantitation of relative fluorescence intensities indicative of relative strengths of mitochondrial membrane potential. Error bars indicate standard deviations of results from four independent experiments.

Next, we wanted to determine if small TbTim knockdown had an effect on other nucleus-encoded mitochondrial proteins. Immunoblot analyses of mitochondrial proteins isolated from TbTim9 RNAi, TbTim10 RNAi, and TbTim8/13 RNAi cells were performed using antibodies for TbTim17, which is a polytopic MIM protein. Antibodies for VDAC, TbAAC, TAO, and mtHsp70 were also used to evaluate their steady-state levels. VDAC is a polytopic MOM protein with β-barrel signals; TbAAC is a polytopic MIM protein with internal targeting signals; TAO is a MIM protein that has an N-terminal presequence; mtHsp70 is a matrix protein with an N-terminal presequence (Fig. 7A). Tubulin was used as a loading control. We observed 55% (±5%) and 32% (±6%) reductions in TbAAC and VDAC steady-state levels due to small TbTim knockdown, respectively (Fig. 7B). This suggests that the mitochondrial import or assembly of TbAAC and VDAC may depend on the small TbTim proteins. Surprisingly, the detectable chemiluminescent signal of TbTim17 was severely reduced by 97% (±2%) within 4 days postinduction of RNAi of any of the small TbTims (Fig. 7B). TbTim17 steady-state levels in whole-cell lysates were also significantly reduced in small TbTim RNAi parasites (Fig. S6A). This severe TbTim17 reduction was not due to a reduction in TbTim17 mRNA, as we observed that TbTim17 mRNA levels were unaffected by small TbTim knockdown (Fig. 7C). The levels of these mitochondrial proteins in the uninduced RNAi cells were similar to those in the wild-type control (Fig. S5B). We did not observe any significant changes in the steady-state levels of TAO and mtHsp70 within 4 days of induction of small TbTim RNAi, possibly due to a longer half-life of these proteins. However, we observed a significant reduction in the levels of these proteins upon longer induction (6 days) of small TbTim RNAi (Fig. S6B and C), which was expected because the import of these proteins into mitochondria depends on TbTim17 (36, 38, 39). To determine if small TbTim knockdown had any effect on mitochondrial membrane potential, we did MitoTracker Red staining of wild-type and small TbTim RNAi cells. Small TbTim knockdown did not show any significant effect on mitochondrial membrane potential (Fig. 7D and E) within 4 days after induction of RNAi. Therefore, we conclude that the reduction in protein steady-state levels was not due to a reduction in mitochondrial membrane potential or to damaged mitochondria.

10.1128/mSphere.00204-18.6 FIG S6  Effect of small TbTim knockdown on TbTim17 steady-state levels in whole-cell lysates and the effect of prolonged knockdown on other mitochondrial proteins in mitochondria. (A) Western blot analysis of total cellular proteins isolated from wild-type, TbTim9 RNAi, TbTim10 RNAi, and TbTim8/13 RNAi cells. The cells were grown in the presence of doxycycline (1 µg/ml) for 96 h. Total cellular lysates from approximately 5 × 10^6^ cells were analyzed by SDS-PAGE and immunoblot analysis using the TbTim17 antibody. Tubulin was used as a loading control. (B) Western blot analysis of mitochondrial proteins (50 µg, 25 µg, and 12.5 µg in decreasing order as indicated by the right-pointing triangles) isolated from wild-type, TbTim9 RNAi (TbTim9 KD), TbTim10 RNAi (TbTim10 KD), and TbTim8/13 RNAi (TbTim8/13 KD) cells with the indicated antibodies. The RNAi cells were grown in the presence of doxycycline (1.0 µg/ml) for 144 h. (C) Densitometric analysis of the immunoblots. The intensities of the indicated protein signals for wild-type mitochondria were normalized to the tubulin signal. The normalized values for the wild-type sample were set to 100% (1.0). Error bars indicate standard deviations of results from three independent experiments. Download FIG S6, PDF file, 0.5 MB. Copyright © 2018 Smith et al. 2018 Smith et al. This content is distributed under the terms of the Creative Commons Attribution 4.0 International license.

### Small TbTim knockdown reduces the levels of the TbTim17 complexes.

**FIG 8:**
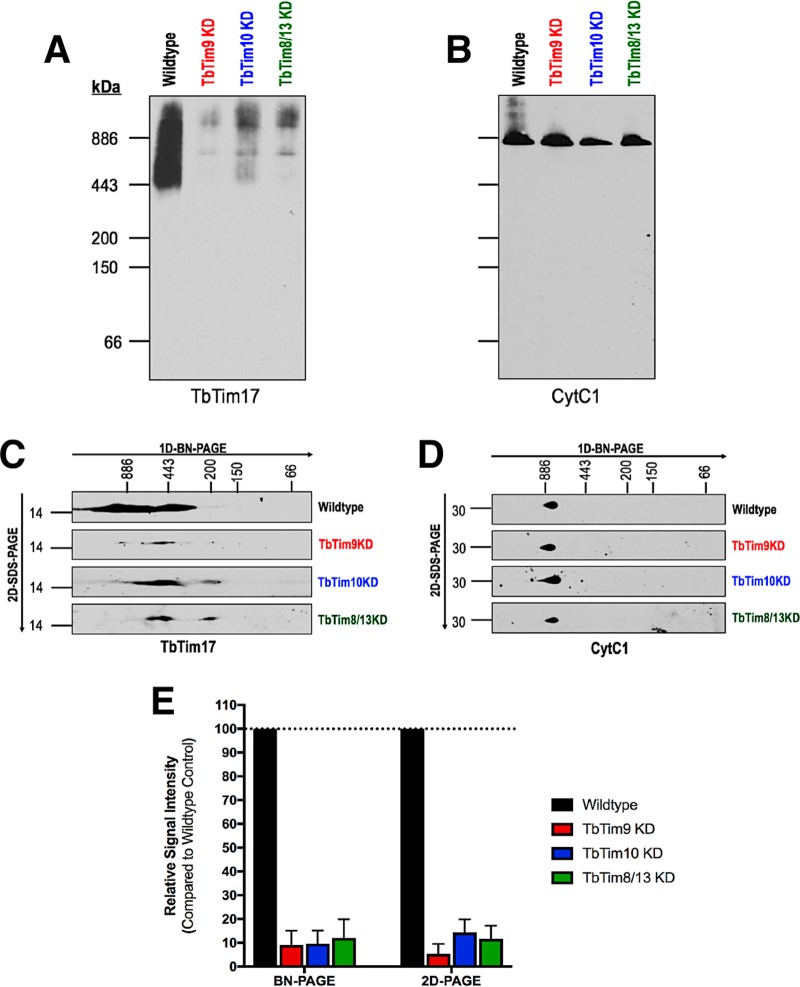
Effect of small TbTim knockdown on the TbTim17 complexes in mitochondria. **(**A and B) Mitochondria isolated from wild-type and small TbTim RNAi cells were solubilized with digitonin, and protein complexes were analyzed by blue native PAGE followed by Western blotting with (A) anti-TbTim17 and (B) anti-CytC1 antibodies. (C and D) Two-dimensional BN/SDS-PAGE (2D-PAGE) analysis of mitochondrial extracts (100 µg) collected from wild-type, TbTim9 RNAi (TbTim9 KD), TbTim10 RNAi (TbTim10 KD), and TbTim8/13 RNAi (TbTim8/13 KD) cells grown in the presence of doxycycline (1.0 µg/ml) for 96 h. Blots were probed with (C) anti-TbTim17 and (D) anti-CytC1 antibodies. (E) Densitometric analysis of the TbTim17 signal intensities from blue native PAGE blots and 2D-PAGE blots normalized to their respective CytC1 signals.

Next, we compared the levels of TbTim17 protein complexes in wild-type and small TbTim knockdown mitochondria by BN-PAGE followed by immunoblot analysis. Anti-TbTim17 antibody recognized TbTim17 protein complexes within a range of ~400 to 1,100 kDa from the wild-type mitochondrial sample as shown previously (38) (Fig. 8A). However, the levels of these complexes were significantly reduced in the small TbTim knockdown mitochondria (Fig. 8A and E). The levels of the respiratory complex III were unaffected (Fig. 8B). To confirm these results, we also separated these complexes by two-dimensional BN–SDS-PAGE and detected the results by immunoblot analysis using the same antibodies. Results further showed that the levels of TbTim17 complexes were significantly reduced in the small TbTim knockdown mitochondria (Fig. 8C and E). Furthermore, we observed smaller TbTim17 subcomplexes in small TbTim knockdown mitochondria (Fig. 8C). This suggests that the TbTim17 complexes were destabilized by small TbTim knockdown. The levels of respiratory complex III were unaffected due to small TbTim knockdown (Fig. 8D).

### Small TbTims are critical for the biogenesis of TbTim17 protein complex.

**FIG 9:**
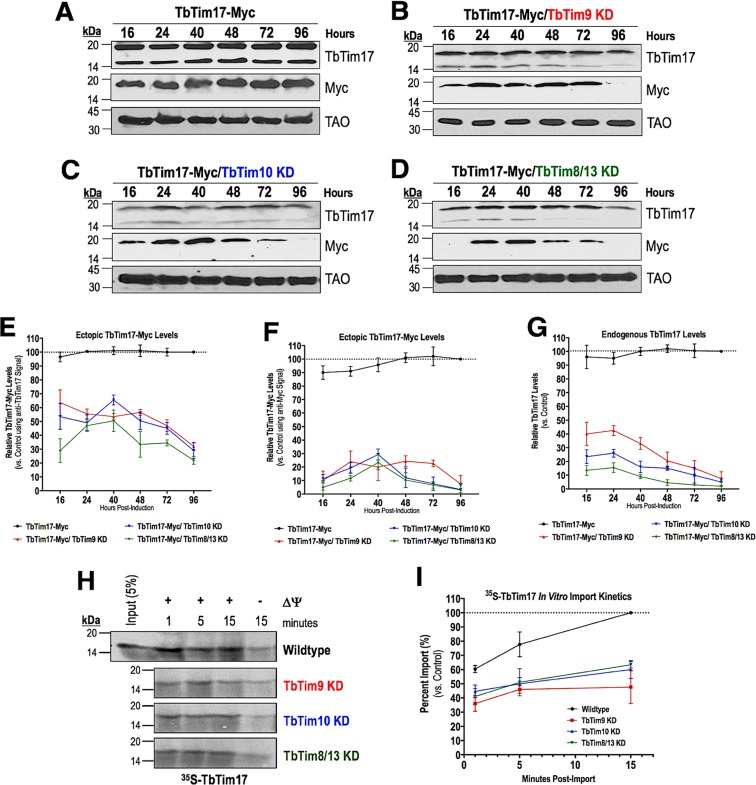
Effect of small TbTim knockdown on import of TbTim17 into mitochondria. **(**A to D) *In vivo* import assays for TbTim17-Myc in (A) control, (B) TbTim9 RNAi, (C) TbTim10 RNAi, and (D) TbTim8/13 RNAi T. brucei mitochondria. Mitochondrial proteins were analyzed by Western blots at different time points from h 16 to h 96 using anti-myc and anti-TbTim17 antibodies. TAO was used as a loading control. (E to G) Quantitation of the levels of ectopic TbTim17-Myc using the (E) anti-TbTim17 antibody and (F) anti-Myc antibody and (G) endogenous TbTim17 in all mitochondrial samples during the time course. Intensities of the ectopic TbTim17-Myc and endogenous TbTim17 bands were normalized with the levels of TAO in the corresponding sample. Relative band intensities are presented as percentages of maximum levels of ectopic TbTim17-Myc or endogenous TbTim17 in control mitochondria at 96 h (considered to represent 100%). Error bars indicate standard deviations of results from three independent experiments. (H) Autoradiograph of the *in vitro* import of radiolabeled TbTim17 in wild-type and small TbTim knockdown mitochondria from min 1 to min 15. Mitochondria were also pretreated with CCCP to disrupt membrane potential (ΔΨ). (I) Quantitation of imported TbTim17 at various time points. The wild-type signal at 15 min was set to 100. Results represent three independent experiments.

We have shown previously that ectopically expressed TbTim17-Myc entered into mitochondria and assembled in the endogenous TbTim17 protein complexes in T. brucei (38). Therefore, in order to assess the effect of small TbTim knockdown on the biogenesis of TbTim17 protein complexes, we inducibly expressed TbTim17-Myc in wild-type and small TbTim knockdown mitochondria and determined the kinetics (16 h to 96 h postinduction) of the accumulation of TbTim17-Myc in the mitochondrial fractions by immunoblot analysis (Fig. 9A to D). As observed previously (38), anti-TbTim17 polyclonal antibody recognized both the ectopic TbTim17-Myc and the endogenous TbTim17 whereas the anti-myc antibody recognized only the ectopically expressed TbTim17-Myc. We found that the anti-TbTim17 antibody recognized TbTim17-Myc consistently in the mitochondrial fractions throughout the period, except for the 96-h time point, indicating that TbTim17-Myc was targeted to mitochondria in TbTim9 RNAi, TbTim10 RNAi, and TbTim8/13 RNAi cells. However, TbTim17-Myc levels in mitochondria, as detected by anti-myc antibody, were markedly reduced after 48 h, particularly in TbTim10 RNAi and TbTim8/13 RNAi cells (Fig. 9B to D). This discrepancy at the levels of TbTim17-Myc, as detected by anti-TbTim17 and anti-myc antibodies, is most likely due to differential sensitivity of these antibodies for TbTim17-Myc. In contrast to the small TbTim knockdown mitochondria, TbTim17-Myc consistently accumulated throughout the time periods (16 to 96 h) in the wild-type mitochondria as detected by both antibodies (Fig. 9A). In addition, the overall levels of TbTim17-Myc were about 40% to 70% lower in the RNAi cells than in the wild-type cells throughout the time course, even at earlier time points (16 to 24 h), as detected by the anti-TbTim17 antibody (Fig. 9E). Analyzing the kinetics of TbTim17-Myc levels during the time course using the anti-myc signal, TbTim17-Myc levels in the mitochondria were noticeably lower (Fig. 9F). Interestingly, we observed that the endogenous mitochondrial levels of TbTim17 were quickly reduced at as early as 16 to 24 h and further decreased over time due to the depletion of TbTim9, TbTim10, or TbTim8/13 (Fig. 9G), suggesting that the stability of TbTim17 is greatly compromised in small TbTim knockdown mitochondria. We also performed *in vitro* import assays using radiolabeled TbTim17 and mitochondria isolated from the wild-type and small TbTim knockdown mitochondria. We found that TbTim17 was imported into small TbTim knockdown mitochondria; however, the overall amount of TbTim17 imported within 15 min was lower in small TbTim knockdown mitochondria than in wild-type mitochondria (Fig. 9H and I). As expected, TbTim17 import in all samples was inhibited more when mitochondrial membrane potential was inhibited. Therefore, our *in vitro* import results were well correlated with the targeting of the ectopic TbTim17-Myc into mitochondria in T. brucei. Taking the data together, it appears that small TbTim knockdown partially inhibits the import of TbTim17 and additionally reduces the stability of TbTim17 in T. brucei mitochondria.

## DISCUSSION

We report here for the first time the functions of TbTim9, TbTim10, and TbTim8/13. We showed that TbTim9, TbTim10, and TbTim8/13 are divergent functional homologues of eukaryotic small Tim proteins in T. brucei mitochondria. In spite of the absence of a recognizable TIM22 complex in T. brucei, the small TbTims, especially TbTim8/13, are necessary for optimal parasite growth. TbTim9, TbTim10, and TbTim8/13 associate with each other and associate with TbTim17. Depletion of any of the small TbTims severely reduced the levels of TbTim17 in mitochondria. Importantly, these small TbTims are crucial for the biogenesis of the TbTim17 complexes in T. brucei.

Although the small TbTims are relatively small proteins, they are found in large protein complexes. It is likely that a significant fraction of these three small TbTims associates with TbTim17 in a stable manner, much as human Tim10b or fungal Tim12 stably associates with the TIM22 complex (28, 52). TbTim17 is the only protein homologue of the Tim17/Tim22/Tim23 protein family in T. brucei (51, 53) and is involved in the import of proteins that have either an N-terminal presequence or internal signals targeting mitochondria (36, 39, 51), thus most likely serving the functions of both TIM22 and TIM23 complexes in other eukaryotes (36, 39). In yeast, the Tim9-Tim10 complex is found mainly as a soluble complex and acts as a *trans*-receptor to bind import substrates that enter mitochondria through the TOM channel (20). In T. brucei, it is possible that portions of these small TbTims exist in the IMS as soluble chaperone complexes as a consequence of interacting with each other. Indeed, we found that TbTim10-Myc is present in two smaller complexes (~150 kDa and <66 kDa). A recent report also showed complexes of similar sizes formed by TbTim11-HA, TbTim12-HA, and TbTim13-HA, and by the endogenous TbTim9 and TbTim12 (40). However, we could not detect complexes of similar sizes for either TbTim9-Myc or TbTim8/13-Myc, which could have been due to lower expression levels of these proteins than of TbTim10-Myc. Further studies are needed to clarify the composition of the soluble small TbTim complex(es) in T. brucei.

Using yeast two-hybrid analysis, we found that the small TbTims are capable of interacting with each other. The results showed that the interaction of TbTim8/13 with TbTim9 and TbTim10 was stronger than the interaction between the latter two small TbTims, suggesting that TbTim8/13 could be the central protein needed to form a ternary small TbTim complex. It has been shown previously that, expressed in bacteria, Tim9 and Tim10 of yeast are folded correctly and associate with each other to form a complex similar to the native Tim9-Tim10 hexameric complex (29, 54, 55). Therefore, it is likely that these small TbTims folded properly during expression in yeast. We also verified that the small TbTim fusion proteins are expressed in yeast. However, further experiments are required to confirm whether this unique interaction pattern of the small TbTims holds true in T. brucei mitochondria.

We have found that knockdown of TbTim8/13 caused cell growth to cease, whereas a reduction in the level of TbTim9 or TbTim10 was less detrimental. These different phenotypes could be due to the differential levels of efficiency of the small TbTim RNAi with respect to reducing the levels of their specific target transcripts. It is also possible that TbTim8/13 performs a rate-limiting function that neither TbTim9 nor TbTim10 is able to perform and, therefore, that TbTim8/13 knockdown is more detrimental. However, it is difficult to confirm these results at the protein level due to a lack of specific antibodies. In any case, robust TbTim17 depletion was found in all small TbTim RNAi cell lines. We also noticed that depletion of any of the small TbTims not only reduced the levels of TbTim17 protein complexes but also generated TbTim17 protein complexes of smaller molecular size, showing that these small TbTims are required for the stability of this complex. *In vivo* and *in vitro* import assays revealed a moderate reduction of import of TbTim17 in small TbTim knockdown mitochondria. Using a transgenic cell line, we showed that the rate of decrease of the endogenous TbTim17 is much higher than the rate of import of newly synthesized TbTim17-Myc into mitochondria, which suggests that TbTim17 stability is greatly perturbed due to knockdown of the small TbTims.

It is likely that the small TbTims and TbTim17 together serve as the TIM22-like import pathway in T. brucei. In yeast, import of Tim22 requires the Tim9-Tim10 complex, which carries Tim22 from the TOM complex to the TIM22 complex. In addition to serving as a docking site for the Tim9-Tim10 complex, Tim12 maintains the integrity of the TIM22 complex. Thus, knockdown or inhibition of the small Tims in yeast would quickly cause a defect in Tim22 import and TIM22 complex stability. As a result, Tim22 steady-state levels decrease (28, 56). This is what we suggest happens to TbTim17 in T. brucei when the small TbTims are reduced by RNAi. Knockdown of any of these three small TbTims impairs the integrity of the TbTim17 complex, which causes destabilization of this complex and a severe reduction in TbTim17 steady-state levels. Taken together, our findings demonstrate the importance of TbTim9, TbTim10, and TbTim8/13 in mitochondrial biogenesis of TbTim17 and in cell survival for procyclic parasites.

## MATERIALS AND METHODS

### Protein structure homology modeling.

The primary amino acid sequences of TbTim9 (Tb927.7.2200), TbTim10 (Tb927.3.1600), and TbTim8/13 (Tb927.11.5390) were entered into the SWISS-MODEL ExPasy Web server to generate predicted tertiary structures. No protein templates were suggested. The top three best-fit models, according to the global model quality estimation (GMQE) numbers, were used. GMQE numbers range from 0 (least reliable) to 1 (most reliable). Homology models were based on available crystal structures in the Protein Database Base (PDB).

### Cell maintenance, growth medium, and cell growth analysis.

The procyclic form of the T. brucei 427 doubly resistant cell line (29-13) expressing a tetracycline repressor gene and a T7 RNA polymerase was grown in SDM-79 medium supplemented with 10% fetal bovine serum, G418 (15 µg/ml), and hygromycin (50 µg/ml) (57). To measure cell growth, cells were seeded at a cell density of 2 × 10^6^ cells/ml in fresh medium containing the appropriate antibiotics. Cells were harvested at different time points (up to 10 days), and cells were counted using a Neubauer hemocytometer. The log of cumulative cell numbers was plotted versus time (in days) of incubation. T. brucei 29-13 cells served as wild-type parasites.

### Generation of plasmid constructs and T. brucei transgenic cell lines.

For generation of TbTim9-Myc, TbTim10-Myc, or TbTim8/13-Myc expression constructs, the open reading frames (ORFs) of TbTim9, TbTim10, or TbTim8/13 were subjected to PCR amplification using T. brucei 427 genomic DNA as the template and the corresponding sequence-specific primers (see Table S4 in the supplemental material). TbTim17 (Tb927.11.13290) was previously cloned in the same manner (51, 58). The forward and reverse primers were designed to add restriction sites for HindIII and XbaI at the 5′ and 3′ ends of the ORFs, respectively. The PCR products were cloned into the pLew100-Myc vector within the HindIII and XbaI restriction sites (59–61). Plasmid DNA was linearized by NotI digestion and transfected into T. brucei 29-13 cells as previously described (61). Transfected cells were selected with phleomycin (2.5 µg/ml). The TbTim9 RNAi, TbTim10 RNAi, and TbTim8/13 RNAi constructs were generated by PCR amplification of the ORFs of TbTim9, TbTim10, or TbTim8/13 using T. brucei genomic DNA as the template and sequence-specific primers (Table S4). The forward and reverse primers were designed to add restriction sites for HindIII and BamHI at the 5′ and 3′ ends of the ORFs, respectively. The PCR products were cloned into tetracycline-inducible T7 double-headed promoter p2T7^Ti^-177 RNAi vector between the HindIII and BamHI restriction sites (62). Plasmid DNA was linearized by NotI digestion and transfected into T. brucei 29-13 cells. Transfected cells were selected with phleomycin (2.5 µg/ml). We also generated small TbTim RNAi constructs using p2T7^Ti^-177 RNAi (puromycin) vector. Total transfected cell populations that were resistant to selective antibiotics after several subcultures were used for this study. We then transfected the TbTim17-Myc cells that had been established previously in the laboratory (51, 58) with the puromycin-selective small TbTim RNAi constructs for generation of small TbTim RNAi cells that simultaneously expressed the double-stranded RNA for the specific small TbTim and TbTim17-Myc transcript.

10.1128/mSphere.00204-18.10 TABLE S4  Primers used in this study. The primers used for the generation of the small TbTim overexpression cell lines (TbTim9-Myc, TbTim10-Myc, and TbTim8/13-Myc) and small TbTim RNAi cell lines (TbTim9 RNAi, TbTim10 RNAi, and TbTim8/13 RNAi) and for quantitative reverse transcriptase PCR (TbTim9 qRT-PCR, TbTim10 qRT-PCR, TbTim8/13 qRT-PCR, TbTim17 qRT-PCR, and tubulin qRT-PCR) are shown in the table. Both forward and reverse primers are shown. All DNA sequences are written from 5′ to 3′. Restriction enzyme sites are underlined, and start codons are bolded. Download TABLE S4, PDF file, 0.2 MB. Copyright © 2018 Smith et al. 2018 Smith et al. This content is distributed under the terms of the Creative Commons Attribution 4.0 International license.

### Isolation and postisolation treatment of mitochondria.

T. brucei parasites were seeded at a cell density of 2 × 10^6^ cells/ml in an Erlenmeyer flask with a 2-liter capacity under conditions of constant agitation at room temperature and allowed to grow for 2 to 4 days. Mitochondria were isolated from the parasites after lysis in isotonic buffer using a nitrogen cavitation bomb (minibomb cell disruption chamber; Kontes, Vineland, NJ) (63, 64). Briefly, cells were washed with 1× phosphate-buffered saline (PBS) and resuspended at a density of 1 × 10^9^ cells/ml in SEMP buffer [250 mM sucrose, 2 mM EDTA, 10 mM 3-(N-morpholino)propanesulfonic acid (MOPS)–KOH (pH 7.2), 2 mM phenylmethylsulfonyl fluoride (PMSF)]. The cells were equilibrated at 700 lb/in^2^ N_2_ for 15 min in a nitrogen cavitation bomb. Subsequent release from the bomb resulted in the disruption of more than 90% of cells. The lysed cell suspension was diluted with 1 vol of the SEMP buffer, and the mitochondrial fraction was isolated by differential centrifugation. The isolated mitochondria were stored at a protein concentration at 10 mg/ml in SEMP buffer containing 50% glycerol at −70°C. For sodium carbonate extraction, mitochondria (100 µg) were incubated with 100 µl of 100 mM sodium carbonate (pH 11.5) on ice for 30 min. The lysate was centrifuged at 14,000 × *g*, and the supernatant and pellet fractions were collected for further analysis.

### Yeast two-hybrid analysis.

The ORFs of TbTim9, TbTim10, and TbTim8/13 were subcloned into yeast expression vectors pGADT7 and pGBKT7 (Clontech) to generate the bait and prey plasmids (65, 66). Approximately 2 µg of each of the bait and prey plasmids in different combination pairs was cotransformed into the Saccharomyces cerevisiae Y2H Gold strain (Clontech; catalog no. 630489) using the lithium acetate method (66). Cotransformed yeast cells were plated on SD medium lacking leu and trp and allow to grow for 3 days at 30°C. Yeast clones that grew were then plated on SD –leu/–trp/–his medium that was lacking leucine, tryptophan, and histidine to select for protein-protein interactions among the small TbTim proteins. SD –leu/–trp/–his plates were also supplemented with 2.0 to 5.0 mM AT, which inhibits Y2H yeast cell growth due to leaky expression of the *HIS3* gene (67), to limit the occurrence of false positives. Inoculated plates were allowed to grow at 30°C for 3 to 5 days. To confirm positive readouts, this process was repeated at least three times with multiple clones.

### RNA isolation and quantitative RT-PCR analysis.

RNA was isolated from T. brucei cells using an RNeasy miniprep isolation kit (Qiagen) and digested with amplification-grade DNase (1 U/µl) for 1 h before first-strand cDNA synthesis was performed using an iScript cDNA synthesis kit (Bio-Rad). The resulting cDNA was amplified using specific primers that recognized the 5′-untranslated region and the 5′ portion of the ORF to detect endogenous TbTim9, TbTim10, TbTim8/13, TbTim17, and tubulin mRNA levels (Table S4) but not the double-stranded RNA generated from the p2T7^Ti^-177 RNAi construct.

### Mitochondrial membrane potential quantitation.

Approximately 2 × 10^7^ T. brucei cells were harvested and incubated in 500 nM MitoTracker Red CMXRos dye (Molecular Probes). Unstained cells and wild-type cells that had been pretreated with 50 µM carbonyl cyanide m-chlorophenyl hydrazone (CCCP) for 15 min before the addition of the MitoTracker Red dye were used as controls. Cells were then fixed by incubation with 0.37% paraformaldehyde. Stained and fixed cells were resuspended in 1× PBS and were analyzed using a FACSCalibur instrument, and emission at 599 nm was quantitated after excitation at 578 nm.

### SDS-PAGE and immunoblot analysis.

Proteins from whole-cell lysates or cytosolic or mitochondrial extracts were separated on a 12% Tris-SDS polyacrylamide gel, transferred to a nitrocellulose membrane, and immunodecorated with polyclonal antibodies for TbTim17 (Tb927.11.13920) (53), TbAAC (Tb927.10.14820) (53), VDAC (Tb927.2.2510) (68), TbPP5 (Tb927.10.13670) (49), cytochrome *bc*-1 (CytC1) (Tb927.8.1890) (69), RBP16 (Tb927.11.7900) (70), and mtHsp70 (Tb927.6.3740) (71) or monoclonal antibodies for the myc epitope (9E10 clone; Vanderbilt University Molecular Biology Core), TAO (Tb927.10.7090) (72), and T. brucei β-tubulin (Tb927.1.2350) (73). Blots were developed with appropriate secondary antibodies and an enhanced chemiluminescence kit (Pierce).

### Two-dimensional BN/SDS-PAGE analysis (2D-PAGE).

Mitochondrial proteins (200 µg) were solubilized in 100 µl of ice-cold 1× native buffer (Life Technologies, Inc.) containing 1% digitonin. The solubilized mitochondrial proteins were clarified by centrifugation at 100,000 × *g* for 30 min at 4°C. The supernatants were mixed with G250 sample additive (Invitrogen) and were electrophoresed on a precast (4% to 16%) bis-Tris polyacrylamide gel (Invitrogen), according to the manufacturer’s protocol. Protein complexes were detected by immunoblot analysis. Molecular size marker proteins apoferritin dimer (886 kDa) and apoferritin monomer (443 kDa), β-amylase (200 kDa), alcohol dehydrogenase (150 kDa), and bovine serum albumin (66 kDa) were electrophoresed on the same gel and visualized by Coomassie staining. Gel strips representing a single lane on the first-dimension native gel were excised. Gel strips were first incubated in buffer containing 1% SDS and 5% β-mercaptoethanol for 30 min to denature proteins and then electrophoresed on a second dimension using Tricine–SDS-PAGE (12%). After separation, proteins were transferred to a nitrocellulose membrane for Western blot analysis.

### *In vitro* mitochondrial protein import assay.

Radiolabeled ([^35^S]l-methionine) TbTim17 and TbAAC were synthesized using a cell-free transcription-and-translation (TNT) rabbit reticulocyte lysate (Promega). Isolated T. brucei mitochondria (100 µg) were suspended in 90 µl of import buffer [250 mM sucrose, 80 mM potassium chloride, 5 mM magnesium chloride, 5 mM dithiothreitol, 1.0 mg/ml fatty acid-free bovine serum albumin, 10 mM 3-(N-morpholino)propanesulfonic acid (MOPS)–KOH (pH 7.2), 2 mM ATP, 10 mM creatine phosphate, 0.1 mg/ml creatine kinase, 8 mM potassium ascorbate, 200 nM N,N,N′,N’-tetramethylphenylenediamine, 5 mM nicotinamide adenine dinucleotide (NADH)]. The mitochondrial suspension was mixed with 10 µl of radiolabeled precursor protein TNT lysate and incubated at 27°C for up to 15 min. Some mitochondria were pretreated with 50 µM carbonyl cyanide m-chlorophenyl hydrazone (CCCP) for 15 min to deplete mitochondrial membrane potential (−Ψ) before the addition of radiolabeled precursor proteins. Mitochondria were washed twice with SEM buffer to remove excess radiolabeled protein. For TbTim17 and TbAAC protein imports, mitochondria were resuspended in SEM buffer (1 µg/µl) and were treated with PK (20 µg/ml) for 10 min on ice. Postimport fractions were analyzed by SDS-PAGE, transferred to a nitrocellulose membrane, and detected by developing exposed autoradiography films.

### *In vivo* mitochondrial protein import assay.

The stable cell lines containing both the TbTim17-Myc overexpression and small TbTim RNAi constructs were treated with doxycycline (1.0 µg/ml) to simultaneously induce the expression of the double-stranded RNA of the specific small TbTims, and the reporter protein TbTim17-Myc cells without the small TbTim RNAi constructs served as controls. At different time points (up to 96 h) after induction with doxycycline, cells were lysed, and the mitochondrial fractions were isolated. Equal amounts of proteins from the mitochondrial fractions were analyzed by SDS-PAGE and immunoblotting to assess the levels of TbTim17-Myc and endogenous TbTim17 in the mitochondria. TAO was used as a loading control.

### Coimmunoprecipitation assay.

Mitochondria (600 µg) were solubilized in 300 μl of 1× cold native buffer (50 mM [Tris pH 7.2], 50 mM NaCl, 10% [wt/vol] glycerol, 1 mM PMSF, 1 µg/ml leupeptin, 1% digitonin) and incubated on ice for 1 h. The solubilized mitochondria were centrifuged at 100,000 × *g* for 30 min. An aliquot (50 µl) of the supernatant was mixed with 50 µl of 2× Laemmli sample buffer and served as the input sample. The remaining supernatant (~250 µl) was mixed with 25 µl of anti-myc agarose bead slurry (Abcam, Inc.) and allowed to incubate for 12 h at 4°C with constant gentle inversion. The beads were then washed three times in wash buffer (50 mM Tris [pH 7.2], 50 mM NaCl, 10% [wt/vol] glycerol, 1 mM PMSF, 1 µg/ml leupeptin, 0.1% digitonin) to remove nonspecifically bound proteins. The washed beads with bound proteins were resuspended in 50 µl of 1× Laemmli sample buffer and served as the bound, precipitated fraction sample. For mass spectrometry analysis, we scaled up this protocol 5-fold. Immunoprecipitated proteins (100%) were loaded on an SDS-PAGE gel. After the proteins had penetrated 1 cm into the resolving gel, we stained the gel with Coomassie brilliant blue and excised the stained area for further analysis by trypsin digestion and mass spectrometry as described below.

### Mass spectrometry analysis.

Excised protein bands from one-dimensional SDS-PAGE were reduced with 10 mM TCEP [Tris(2-carboxyethyl)phosphine hydrochloride] for 60 min, alkylated with 50 mM iodoacetamide for 60 min, and subjected to in-gel digestion with trypsin (100 ng/sample) overnight. The digested peptides were dried in a Speed-Vac concentrator, resuspended in 5 µl of 0.5% formic acid (FA), and loaded onto a nano-column (100 µm by 12 cm; C_18_ resin) with an Eksigent AS1 autosampler. Peptides were analyzed by the use of an Orbitrap LTQ XL linear ion trap mass spectrometer (Thermo Fisher) in nanospray mode. A 90-min acetonitrile gradient consisting of 5% to 50% buffer B [buffer A, 0.1% FA; buffer B, 0.1% FA–acetonitrile] was executed for liquid chromatography-mass spectrometry (LC-MS) analysis using an Eksigent 1D Plus nano-LC pump online with the MS instrument. MS data acquisition was done in a data-dependent 6-event method (using a survey Fourier transform ion cyclotron resonance mass spectrometer [FTMS] scan [resolution, 30,000] followed by five data-dependent tandem MS [MS/MS] scans for determination of the five consequently most abundant ions). The general mass spectrometric settings were as follows: spray voltage, 2.4 kV; no sheath and no auxiliary gas flow; ion transfer tube temperature, 200°C; collision-induced dissociation (CID) fragmentation (for MS/MS), 35% normalized collision energy; activation false-discovery-rate (FDR [*q*]) value of 0.25; activation time, 30 ms. The minimal threshold for the dependent scans was set to 1,000 counts, and a dynamic exclusion list was used with the following settings: repeat count of 1, repeat duration of 30 s, exclusion list size of 500, and exclusion duration of 90 s. Tandem spectra were searched using PEAKS 8.5 software against the recent NCBI T. brucei database merged with the recent NCBI human database. The search database was concatenated with the reverse sequences of all proteins in the database to allow the determination of FDR. The parameters for database search were as follows: full tryptic digestion; up to 2 missed cleavage sites; 10 ppm for peptide mass tolerance; 0.5 Da for fragment mass tolerance; cysteine carbamidomethylation (+57 Da) as a fixed modification; and methionine oxidation (+16 Da) as a variable modification. Protein matches were preliminary filtered using a −10lgP cutoff value of >20 (for both peptides and proteins), an FDR of <5%, and at least one unique peptide and at least two peptides per protein group. The relative quantification of the identified proteins was performed with the Q module of the PEAKS software pack based on the extracted ion currents of the identified parent ions of unique peptides.

### Statistical analysis.

Scatterplots were made in the GraphPad Prism 7.0a software program to show individual data values. A translucent bar was also included to indicate the mean of the data values, and error bars indicate standard deviations. Multiple Student’s *t* tests were used with the Sidak-Bonferroni method to correct for multiple comparisons to determine statistical significance, where a single asterisk (*) indicates a *P* value of <0.05, two asterisks (**) indicate a *P* value of <0.01, three asterisks (***) indicate a *P* value of <0.001, and four asterisks (****) indicate a *P* value of <0.0001.
