# Supplementary material for: Divergent Small Tim Homologues Are Associated with TbTim17 and Critical for the Biogenesis of TbTim17 Protein Complexes in Trypanosoma brucei
Source: mSphere. 2018 Jun 20;3(3):e00204-18. doi: 10.1128/mSphere.00204-18 (PMC6010621; doi:10.1128/mSphere.00204-18)
Supplement: TABLE S1 [file sph003182572st1.pdf]

Paired Small Tim Comparisons	Similarity (%)	Identity (%)
TbTim9 vs. TbTim10	28.8	15.3
TbTim9 vs. TbTim8/13	31.9	22.3
TbTim10 vs. TbTim8/13	35.2	20.0
TbTim9 vs. ScTim9	38.1	21.6
TbTim9 vs. AtTim9	24.8	16.8
TbTim9 vs. HsTim9	37.9	20.0
**AtTim9 vs. HsTim9**	**45.2**	**32.7**
**ScTim9 vs. HsTim9**	**49.0**	**34.4**
TbTim10 vs. ScTim10	38.3	22.6
TbTim10 vs. AtTim10	34.9	20.2
TbTim10 vs. HsTim10	41.8	25.5
**AtTim10 vs. HsTim10**	**47.5**	**29.3**
**ScTim10 vs. HsTim10**	**52.9**	**32.7**
TbTim8/13 vs. ScTim8	32.4	14.7
TbTim8/13 vs. AtTim8	37.9	19.5
TbTim8/13 vs. HsTim8	33.9	18.3
**AtTim8 vs. HsTim8**	**44.9**	**25.5**
**ScTim8 vs. HsTim8**	**50.5**	**33.3**
TbTim8/13 vs. ScTim13	27.4	14.5
TbTim8/13 vs. AtTim13	30.0	14.5
TbTim8/13 vs. HsTim13	23.5	8.7
**AtTim13 vs. HsTim13**	**58.3**	**40.6**
**ScTim13 vs. HsTim13**	**52.7**	**33.6**

TbTim = *Trypanosoma brucei* small Tim
ScTim = *Saccharomyces cerevisiae* (budding yeast) small Tim
AtTim = *Arabidopsis thaliana* (thale rockcress plant) small Tim
HsTim = *Homo sapiens* (human) small Tim
