# Supplementary figures and images for: Divergent Small Tim Homologues Are Associated with TbTim17 and Critical for the Biogenesis of TbTim17 Protein Complexes in Trypanosoma brucei
Source: mSphere. 2018 Jun 20;3(3):e00204-18. doi: 10.1128/mSphere.00204-18 (PMC6010621; doi:10.1128/mSphere.00204-18)

**TbTim9 Models**

**A**

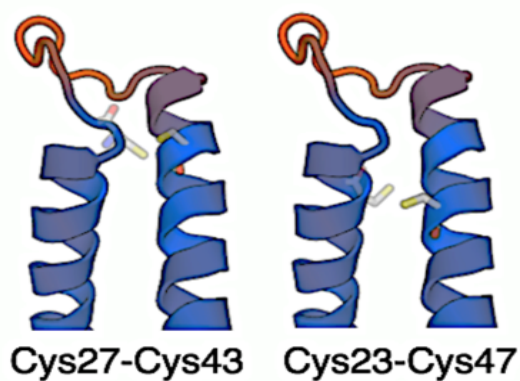

**B**

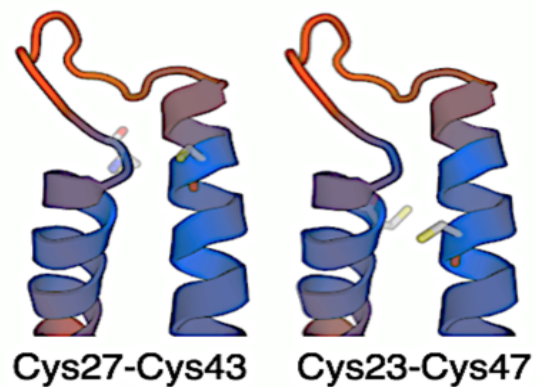

**C**

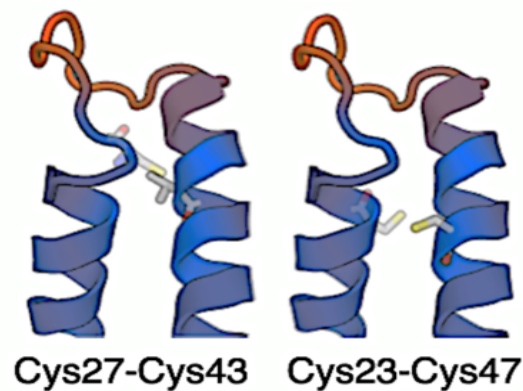

**TbTim10 Models**

**D**

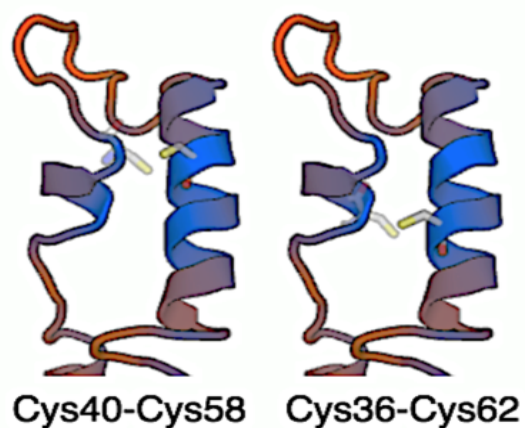

**E**

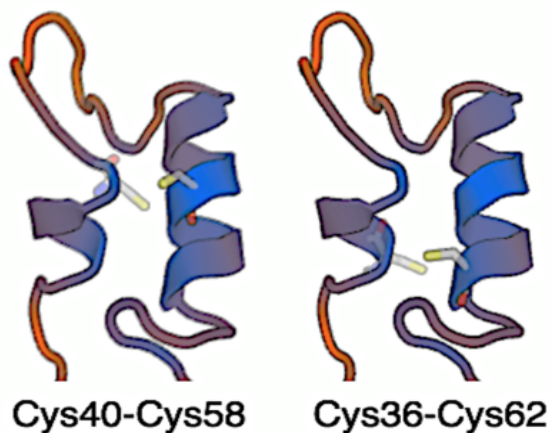

**F**

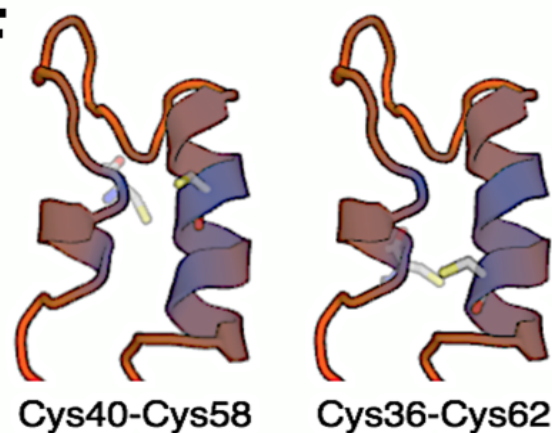

**TbTim8/13 Models**

**G**

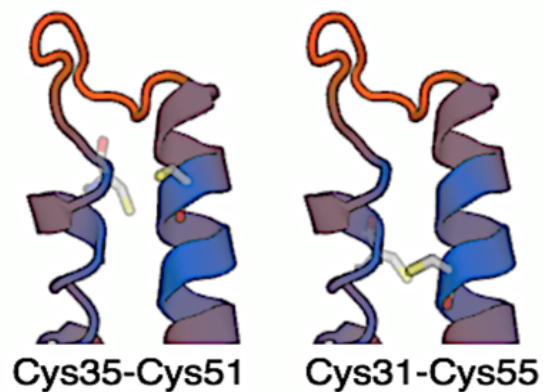

**H**

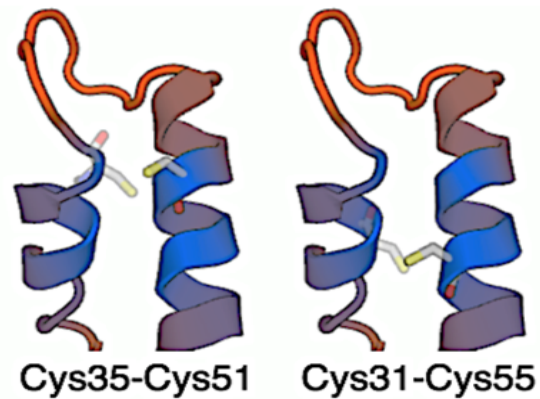

**I**

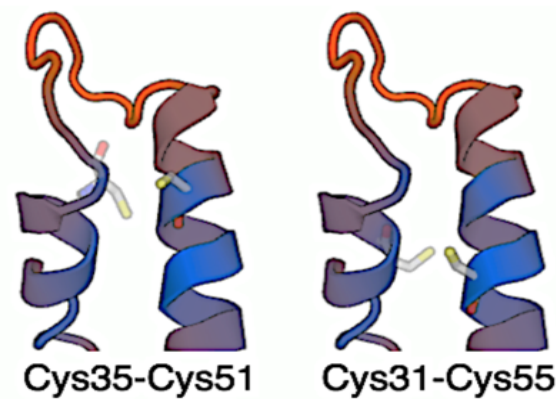

Supplement: FIG S1 [file sph003182572sf1.pdf]

**A**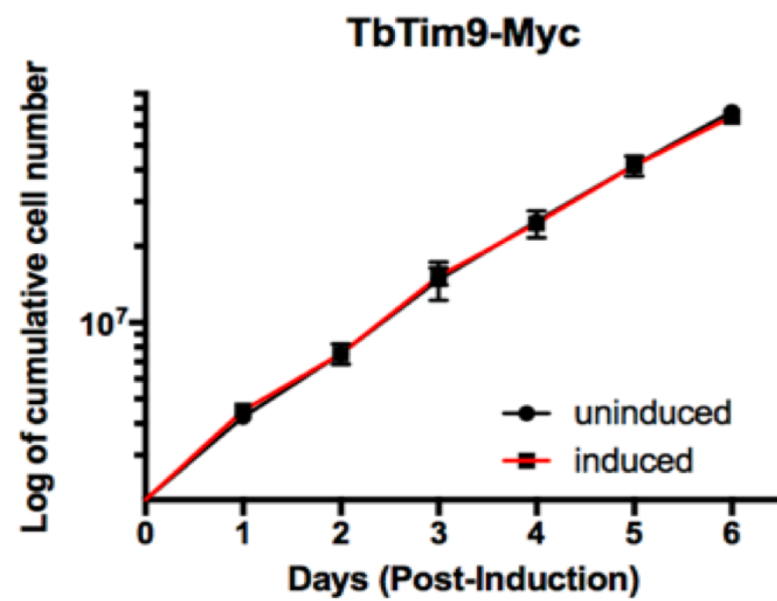**B**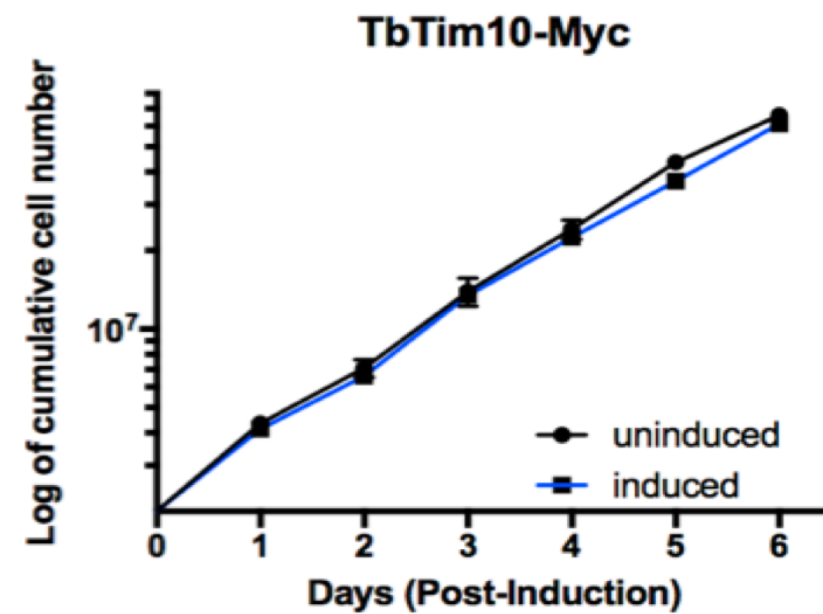**C**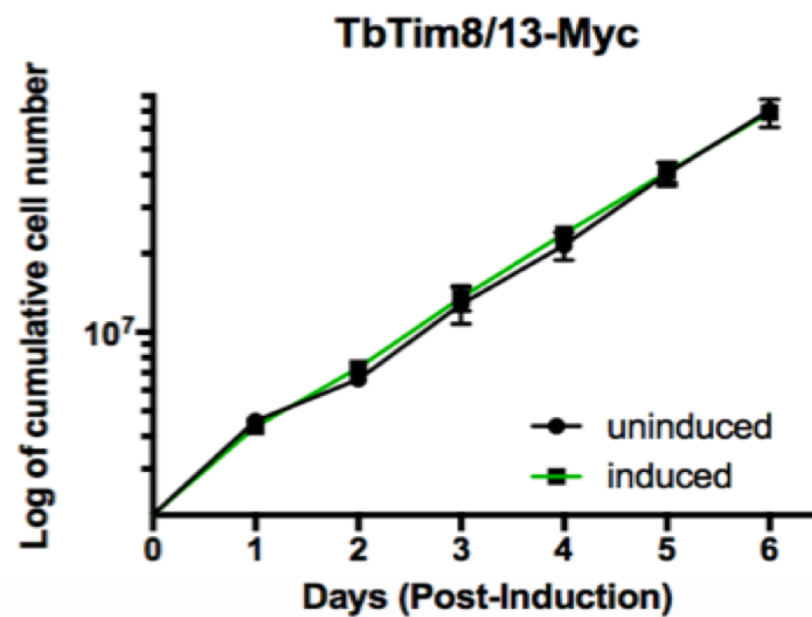

Supplement: FIG S2 [file sph003182572sf2.pdf]

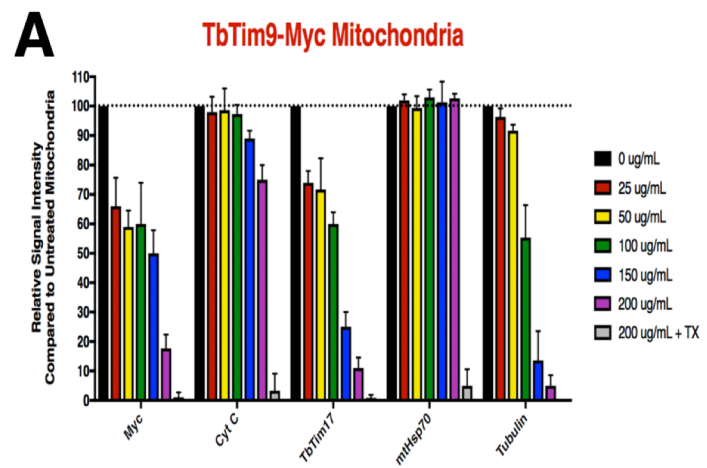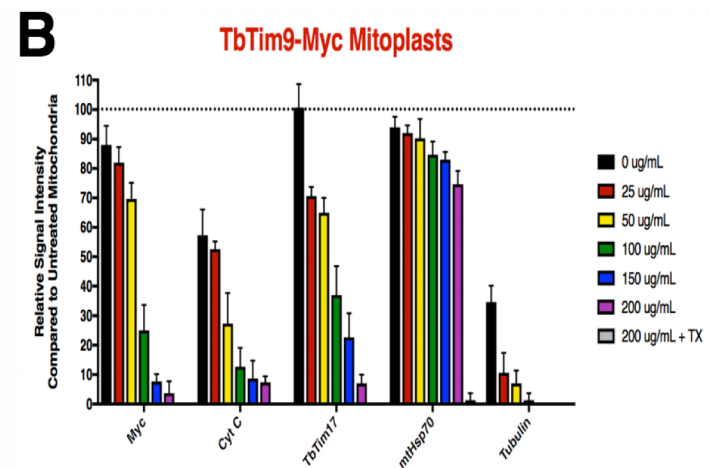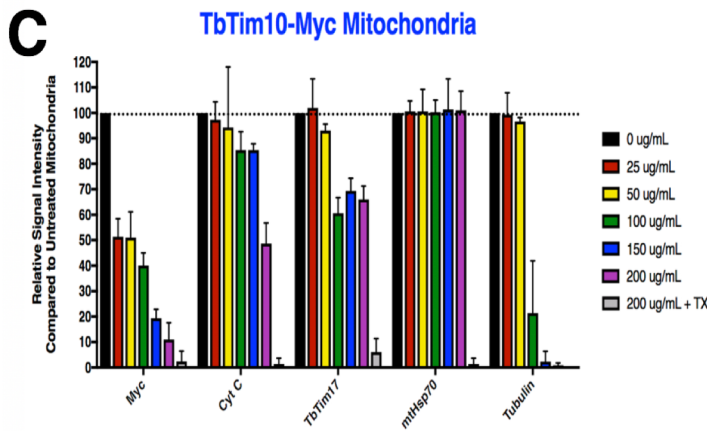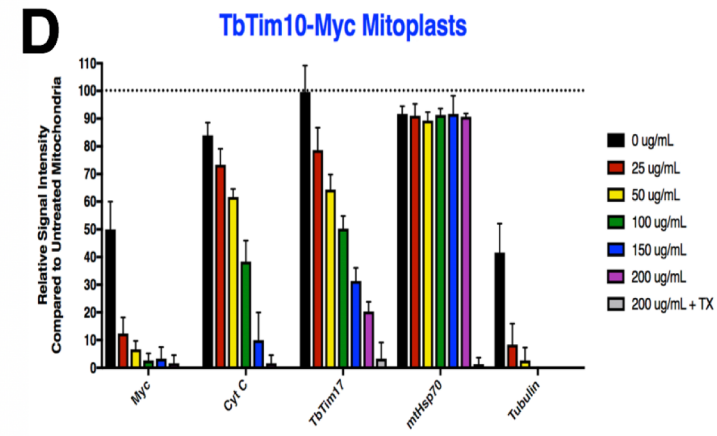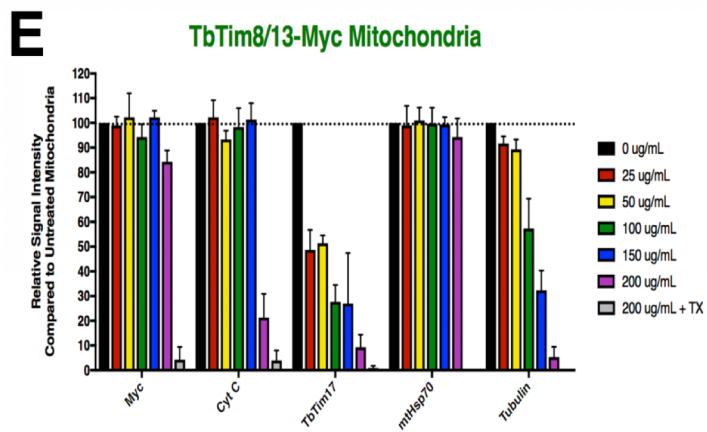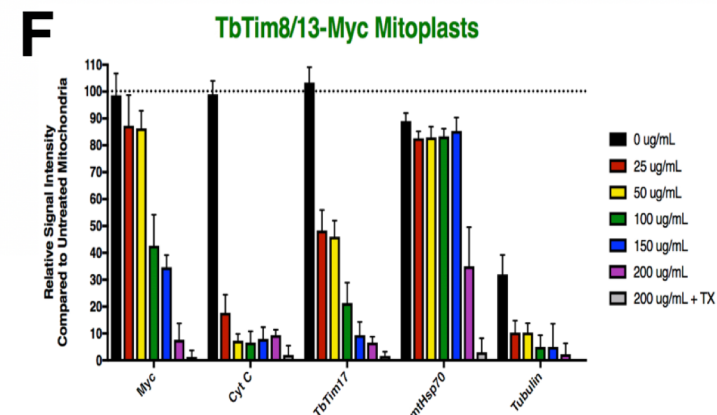

Supplement: FIG S3 [file sph003182572sf3.pdf]

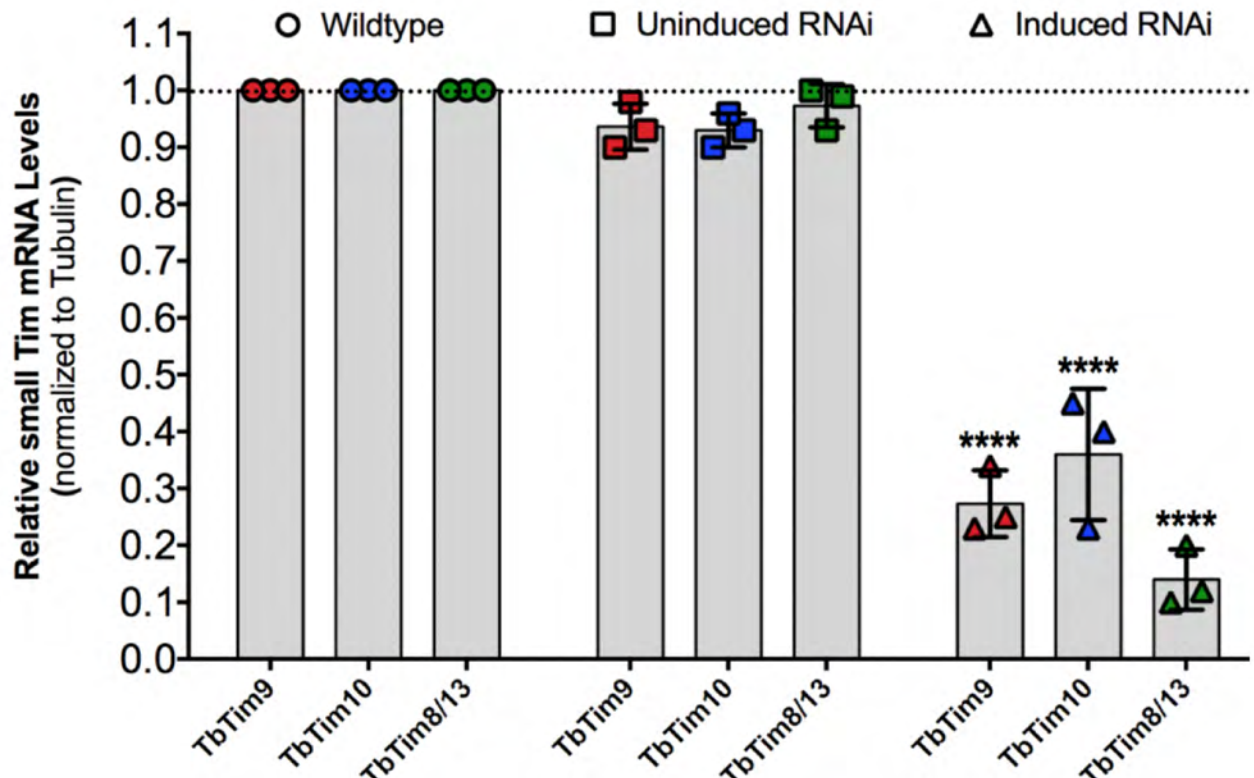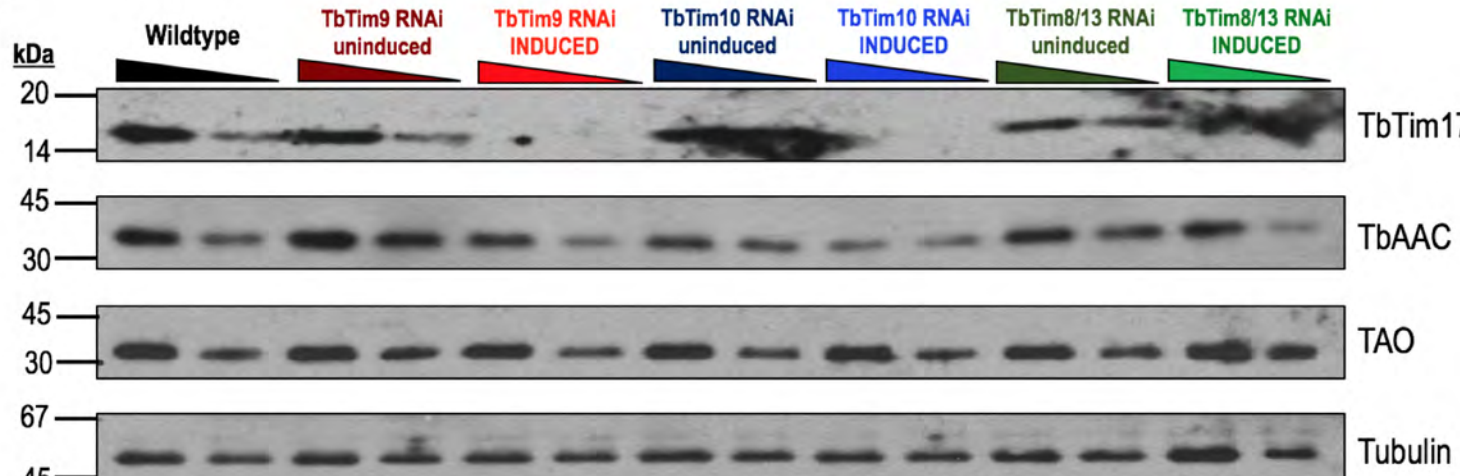

Supplement: FIG S5 [file sph003182572sf5.pdf]

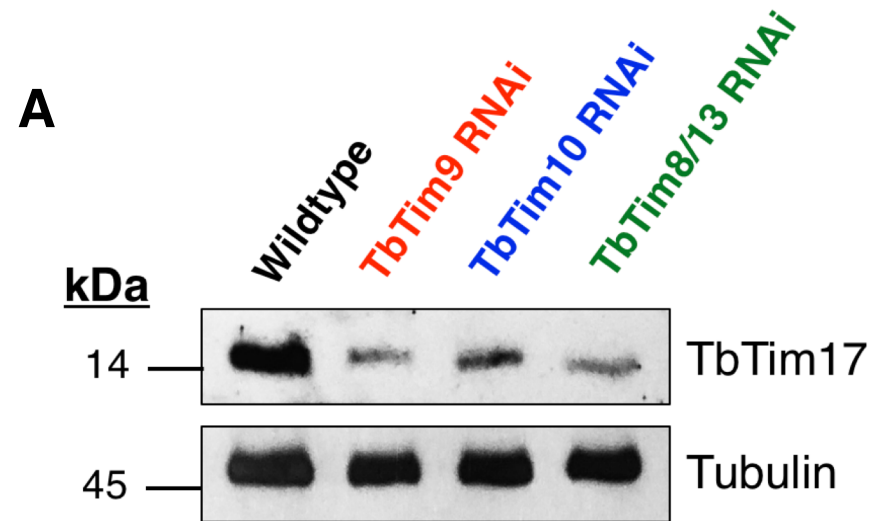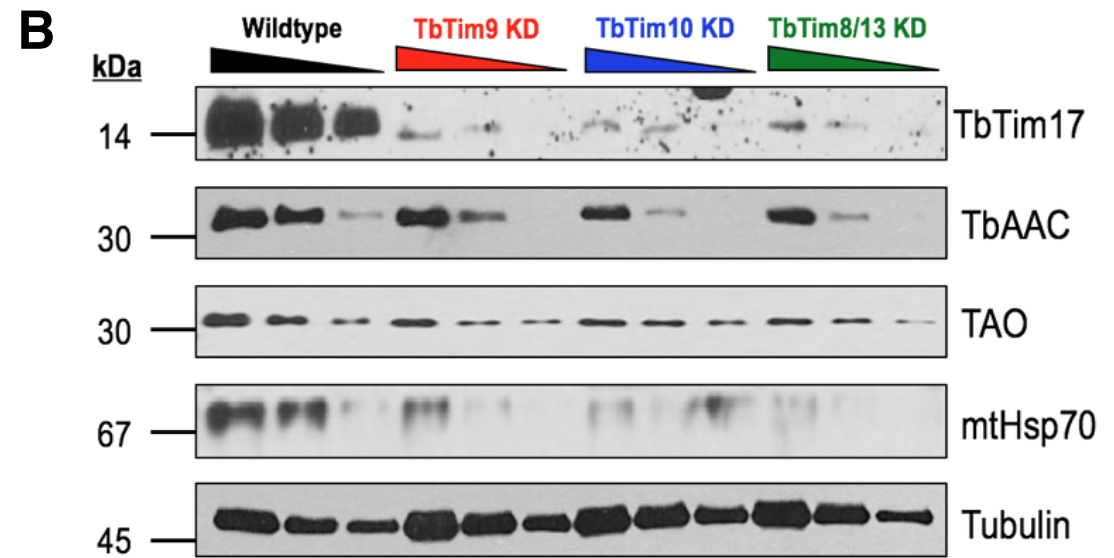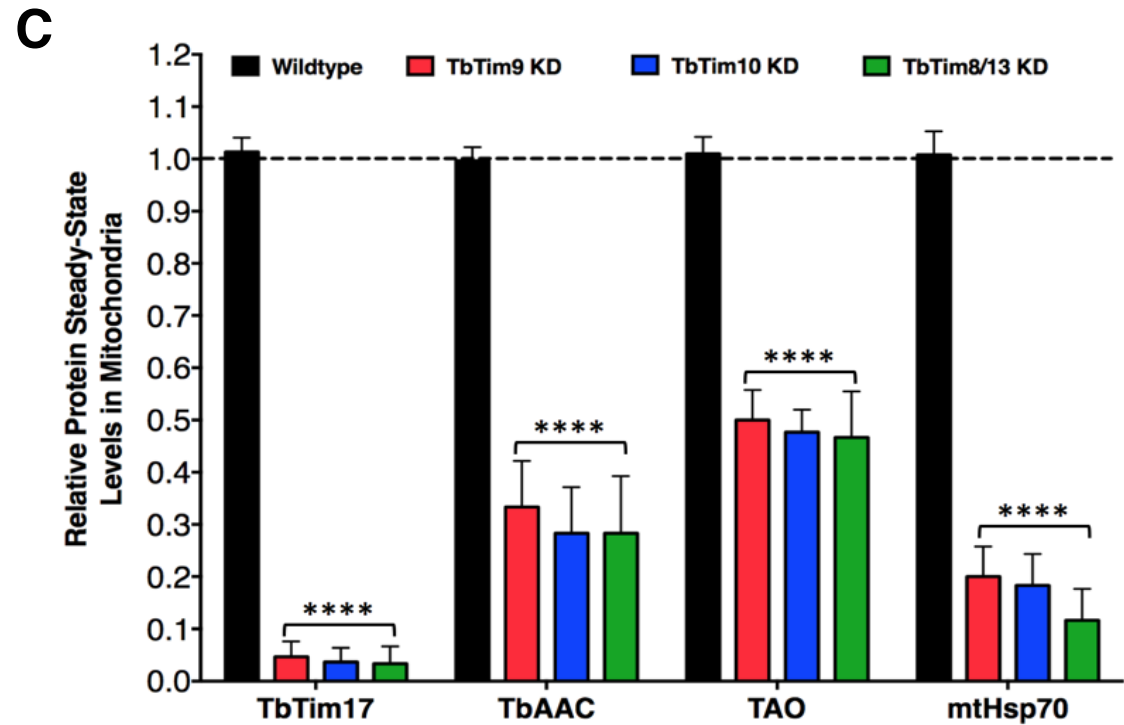

Supplement: FIG S6 [file sph003182572sf6.pdf]
