# Supplementary material for: Divergent Small Tim Homologues Are Associated with TbTim17 and Critical for the Biogenesis of TbTim17 Protein Complexes in Trypanosoma brucei
Source: mSphere. 2018 Jun 20;3(3):e00204-18. doi: 10.1128/mSphere.00204-18 (PMC6010621; doi:10.1128/mSphere.00204-18)
Supplement: TABLE S2 [file sph003182572st2.pdf]

Gene ID[1]	Protein[1]	Coverage (%)	Spectral Intensity	
			Wildtype	TbTim10-Myc
**Tb927.7.2200**	**TbTim9**	81	0	**5.86E+06**
**Tb927.3.1600**	**TbTim10**	58	0	**1.28E+07**
**Tb927.11.5390**	**TbTim8/13**	28	0	**1.20E+06**
**Tb927.5.3340**	**TbTim11**	46	0	**2.24E+06**
**Tb927.4.3430**	**TbTim12**	53	0	**3.04E+06**
**Tb927.10.11520**	**TbTim13**	70	0	**9.76E+06**
**Tb927.11.13290**	**TbTim17**	27	0	**2.50E+06**
**Tb927.9.11220**	**TbTim42**	53	0	**1.89E+07**
**Tb927.8.1740**	**TbTim62**	59	0	**2.18E+07**
**Tb927.8.1420**	**TbACAD**	44	0	**6.58E+06**
Tb927.6.3740	TbHsp70	45	4.76E+05	4.93E+06
Tb927.2.2510	TbVDAC	16	0	2.99E+05
Tb927.9.9660	ATOM40	5	0	1.23E+04
Tb927.6.4280	TbGAPDH	37	3.63E+05	2.24E+06
Tb927.3.1380	TbATPaseβ	22	1.72E+05	2.32E+06
Tb927.10.14830	TbAAC	35	0	4.40E+06
Tb927.10.12840	TbMCP12	29	0	1.02E+06

[1]GeneID as annotated on www.tritrypdb.org.
