# Supplementary material for: Divergent Small Tim Homologues Are Associated with TbTim17 and Critical for the Biogenesis of TbTim17 Protein Complexes in Trypanosoma brucei
Source: mSphere. 2018 Jun 20;3(3):e00204-18. doi: 10.1128/mSphere.00204-18 (PMC6010621; doi:10.1128/mSphere.00204-18)

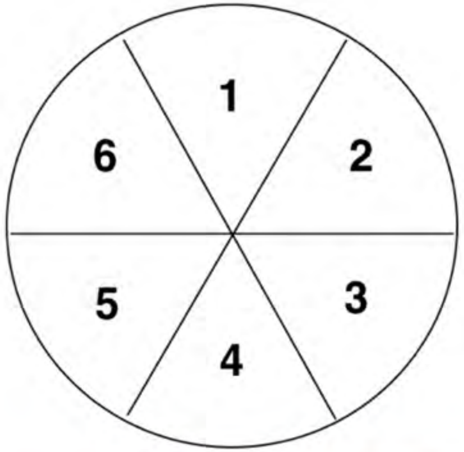

1. SV40-T-AD + p53-BD
2. Empty-AD + Empty-BD (clone 1)
3. Empty-AD alone
4. Empty-BD alone
5. Empty-AD + Empty-BD (clone 2)
6. No DNA

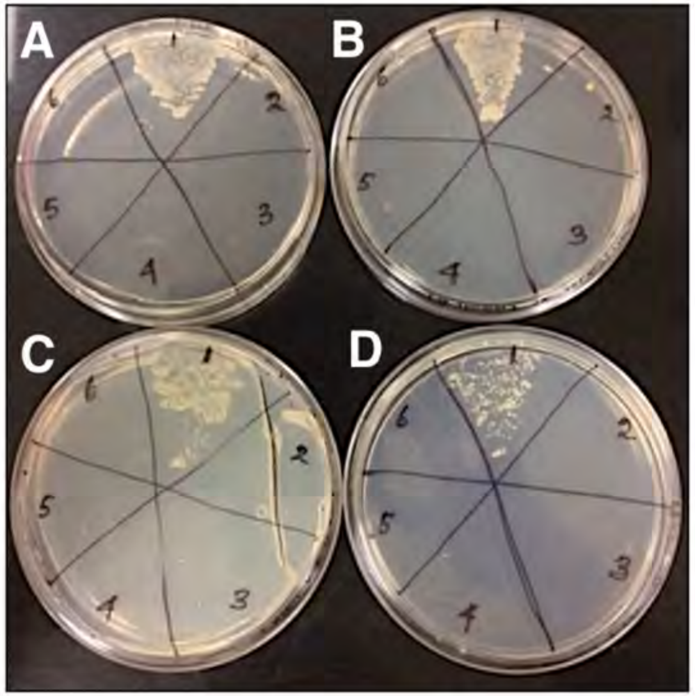

(A) SD –Leu/ –Trp/ –His
(B) SD –Leu/ –Trp/ –His + **2.0 mM AT**
(C) SD –Leu/ –Trp/ –His + **3.5 mM AT**
(D) SD –Leu/ –Trp/ –His + **5.0 mM AT**

E

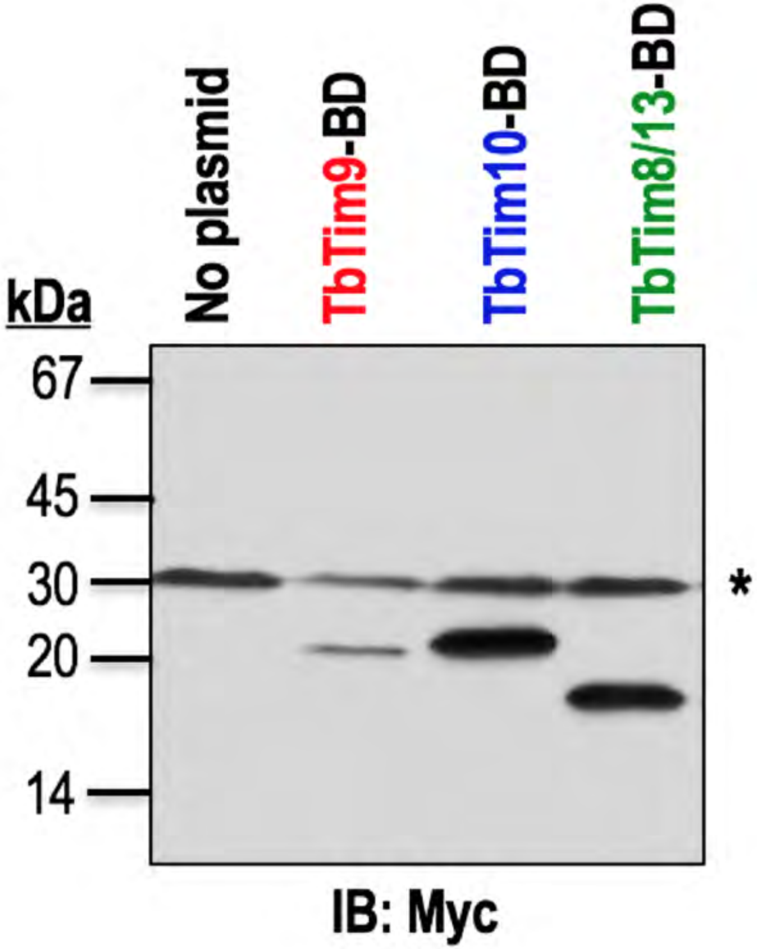

Supplement: FIG S4 [file sph003182572sf4.pdf]
