# Supplementary material for: Divergent Small Tim Homologues Are Associated with TbTim17 and Critical for the Biogenesis of TbTim17 Protein Complexes in Trypanosoma brucei
Source: mSphere. 2018 Jun 20;3(3):e00204-18. doi: 10.1128/mSphere.00204-18 (PMC6010621; doi:10.1128/mSphere.00204-18)
Supplement: TABLE S4 [file sph003182572st4.pdf]

Primer Name	Direction	DNA Sequence* (5' → 3')
TbTim9-Myc	Forward	GATCAAGCTT**ATG**CGCCTGGCTGTAAAG
TbTim9-Myc	Reverse	GATCTCTAGACAACTTCAACATTTG
TbTim10-Myc	Forward	GATCAAGCTT**ATG**CAGCCACCTCAAACG
TbTim10-Myc	Reverse	GATCTCTAGATTCGTTCTCCGACAAAAC
TbTim8/13-Myc	Forward	GATCAAGCTT**ATG**AATCAGTCTAGTTC
TbTim8/13-Myc	Reverse	GATCTCTAGACTTTCCTTTGCTTCC
TbTim9 RNAi	Forward	GATCGGATCCCGCCTGGCTGTAAAG
TbTim9 RNAi	Reverse	GATCAAGCTTCAACTTCAACATTTG
TbTim10 RNAi	Forward	GATCGGATCCCAGCCACCTCAAACG
TbTim10 RNAi	Reverse	GATCAAGCTTTTCGTTCTCCGACAAAAC
TbTim8/13 RNAi	Forward	GATCGGATCCGAATCAGTCTAGTTC
TbTim8/13 RNAi	Reverse	GATCAAGCTTCTTTCCTTTGCTTCC
TbTim9 qRT-PCR	Forward	CCTAAGAACCGTCACCCTTCAAC
TbTim9 qRT-PCR	Reverse	TTTACCGCACAACGCTCCAC
TbTim10 qRT-PCR	Forward	CCATTTGTTGCCCGAGAGTG
TbTim10 qRT-PCR	Reverse	AAGACACGCCTTTTCACCAGG
TbTim8/13 qRT-PCR	Forward	GAGTGGTGCGCTGTATTCATG
TbTim8/13 qRT-PCR	Reverse	CAGTTCCTCATGCAACGGTGC
TbTim17 qRT-PCR	Forward	TGAAGGACAGCACCATTACCCC
TbTim17 qRT-PCR	Reverse	CCGAAAAGGAAACCAAAGTAGGC
Tubulin qRT-PCR	Forward	TTCCGCACCCTGAAACTGA
Tubulin qRT-PCR	Reverse	TGACGCCGGACACAACAG

* Restriction enzyme sites are underlined. Start codons are **bolded**.
